# A Single Regulator Mediates Strategic Switching between Attachment/Spread and Growth/Virulence in the Plant Pathogen *Ralstonia solanacearum*

**DOI:** 10.1128/mBio.00895-17

**Published:** 2017-09-26

**Authors:** Devanshi Khokhani, Tiffany M. Lowe-Power, Tuan Minh Tran, Caitilyn Allen

**Affiliations:** a Department of Plant Pathology, University of Wisconsin—Madison, Madison, Wisconsin, USA; b Microbiology Doctoral Training Program, University of Wisconsin—Madison, Madison, Wisconsin, USA; University of Nebraska-Lincoln

**Keywords:** adhesins, bacterial wilt, dispersal, metabolomics, plant pathogen, quorum sensing, transcriptomics, xylem

## Abstract

The PhcA virulence regulator in the vascular wilt pathogen *Ralstonia solanacearum* responds to cell density via quorum sensing. To understand the timing of traits that enable *R. solanacearum* to establish itself inside host plants, we created a Δ*phcA* mutant that is genetically locked in a low-cell-density condition. Comparing levels of gene expression of wild-type *R. solanacearum* and the Δ*phcA* mutant during tomato colonization revealed that the PhcA transcriptome includes an impressive 620 genes (>2-fold differentially expressed; false-discovery rate [FDR], ≤0.005). Many core metabolic pathways and nutrient transporters were upregulated in the Δ*phcA* mutant, which grew faster than the wild-type strain in tomato xylem sap and on dozens of specific metabolites, including 36 found in xylem. This suggests that PhcA helps *R. solanacearum* to survive in nutrient-poor environmental habitats and to grow rapidly during early pathogenesis. However, after *R. solanacearum* reaches high cell densities *in planta*, PhcA mediates a trade-off from maximizing growth to producing costly virulence factors. *R. solanacearum* infects through roots, and low-cell-density-mode-mimicking Δ*phcA* cells attached to tomato roots better than the wild-type cells, consistent with their increased expression of several adhesins. Inside xylem vessels, Δ*phcA* cells formed aberrantly dense mats. Possibly as a result, the mutant could not spread up or down tomato stems as well as the wild type. This suggests that aggregating improves *R. solanacearum* survival in soil and facilitates infection and that it reduces pathogenic fitness later in disease. Thus, PhcA mediates a second strategic switch between initial pathogen attachment and subsequent dispersal inside the host. PhcA helps *R. solanacearum* optimally invest resources and correctly sequence multiple steps in the bacterial wilt disease cycle.

## IMPORTANCE

*Ralstonia solanacearum* is a destructive soilborne crop pathogen that wilts plants by colonizing their water-transporting xylem vessels. It produces its costly virulence factors only after it has grown to a high population density inside a host. To identify traits that this pathogen needs in other life stages, we studied a mutant that mimics the low-cell-density condition. This mutant (the Δ*phcA* mutant) cannot sense its own population density. It grew faster than and used many nutrients not available to the wild-type bacterium, including metabolites present in tomato xylem sap. The mutant also attached much better to tomato roots, and yet it failed to spread once it was inside plants because it was trapped in dense mats. Thus, PhcA helps *R. solanacearum* succeed over the course of its complex life cycle by ensuring avid attachment to plant surfaces and rapid growth early in disease, followed by high virulence and effective dispersal later in disease.

## INTRODUCTION

Many microbes coordinate their behavior by means of a cell-to-cell signaling system called quorum sensing (QS) (1–4). Quorum sensing is mediated by small diffusible signal molecules that accumulate as microbial populations increase or persist in confined spaces. If the signal reaches a critical concentration inside cells, it changes gene expression. The resulting density-dependent collective behavior ensures that quantitative traits such as production of bioluminescence, antibiotics, and virulence factors are expressed only when enough microbes are present to have a biological effect (2–4). Diverse animal and plant pathogens use QS to upregulate virulence factor expression at high cell densities (2, 3), but less attention has been given to how bacteria use QS to modulate traits that are important for success at low cell densities. Identifying genes that are upregulated at low cell densities may reveal how microbes adapt and protect themselves under unfavorable conditions.

The soilborne plant pathogen *Ralstonia solanacearum* occupies diverse ecological niches. It can survive for years in soil or water as a saprophyte but can also live in plant vascular systems as an aggressive pathogen (5, 6). The bacterium enters host plant through wounds, root elongation zones, or sites of lateral root emergence and colonizes the root cortex to invade its preferred *in planta* habitat, the water-transporting xylem vessels. There *R. solanacearum* rapidly multiplies to high population densities, spreading systemically and producing large amounts of extracellular polysaccharide (EPS), which results in wilting symptoms and, eventually, plant death (7).

*R. solanacearum* uses a sophisticated sensory and regulatory network to transition between its saprophytic and parasitic modes. At the center of this network is the Phc QS system, composed of the LysR-type transcriptional regulator PhcA and the products of the *phcBSR* operon, which control the levels of functional PhcA in response to cell density (8–13).

As noted in the first description of *R. solanacearum* in 1896, this pathogen frequently becomes nonmucoid and avirulent in culture (14). In addition to lacking EPS, these spontaneous mutants have increased motility and aerotaxis (13, 15–17). In 1993, Denny and coworkers discovered that such spontaneous “phenotype converted” (PC) strains resulted from mutation of the *phcA* locus (13). Subsequent studies found that *phcA* mutants are hypermotile and overproduce the siderophore staphyloferrin B but that they make less EPS and plant cell wall-degrading endoglucanase and cellobiohydrolase than the wild type (12, 18, 19). The dysregulation of these multiple virulence factors explains why these mutants are nearly avirulent, although they can still colonize plant stems if introduced through a wound (12).

PhcA directly regulates expression of at least two genes, the endoglucanase structural gene *egl* and the EPS regulator gene *xpsR*, by binding to a conserved 14-bp PhcA box in their promoters (20). Many more traits are indirectly regulated by PhcA via other regulatory loci such as *pehSR*, *vsrAD*, *vsrBC*, *flhDC*, and *solIR* (5, 21). The full extent of the PhcA regulon is unknown, but the many differences between the extracellular protein profiles of wild-type and *phcA* mutant strains of *R. solanacearum* indicate that numerous additional genes are directly or indirectly controlled by this system (22).

Most traits putatively regulated by PhcA have been experimentally verified only *in vitro*, with the important exception of EPS. Immunofluorescence microscopy of *R. solanacearum*-infected tomato plants using anti-EPS antibodies demonstrated that EPS production is dependent on cell density *in planta* (23, 24). Accumulating evidence suggests that *in vitro* studies are not reliable for understanding *R. solanacearum* regulation during plant pathogenesis. For example, PhcA regulates two important bacterial wilt virulence factors differently in culture and *in planta*: swimming motility and the pathogen’s type III secretion system (T3SS). Direct microscopic observation revealed that the percentage of motile cells was density dependent in culture, increasing rapidly as bacteria passed the QS threshold concentration of 10^7^ CFU/ml (9, 25). In contrast, bacteria sampled from xylem vessels were nearly all nonmotile, even when population sizes exceeded 10^9^ CFU/ml xylem fluid (26). Similarly, PhcA repressed the T3SS 20- to 60-fold at high cell densities in culture (27). However, analyses performed with reporter genes, quantitative reverse transcriptase PCR (qRT-PCR), and microarray analyses all found that the T3SS is actually highly expressed during plant infections (28–30). These inconsistencies suggest that the roles of PhcA can be assessed only in the biologically relevant context of a plant host.

To define the full extent of the PhcA QS system and to identify novel virulence factors that are expressed only at low cell densities, we compared the transcriptome of wild-type *R. solanacearum* to that of a Δ*phcA* mutant during colonization of tomato plants. About 12% of the pathogen’s genes were differentially expressed in the Δ*phcA* mutant, with an overall profile suggesting that at low cell densities *R. solanacearum* is optimized to obtain nutrients and attach to plant surfaces. Functional validation experiments confirmed that the Δ*phcA* mutant grew better than the wild type on many specific nutrients and in infected tomato xylem sap. This low-cell-density-mode-mimicking mutant attached better to tomato roots but formed aberrantly dense mats inside xylem vessels and spread poorly in tomato stems.

## RESULTS

### Phenotypes of the low-cell-density-mimicking Δ*phcA* mutant.

**FIG 1:**
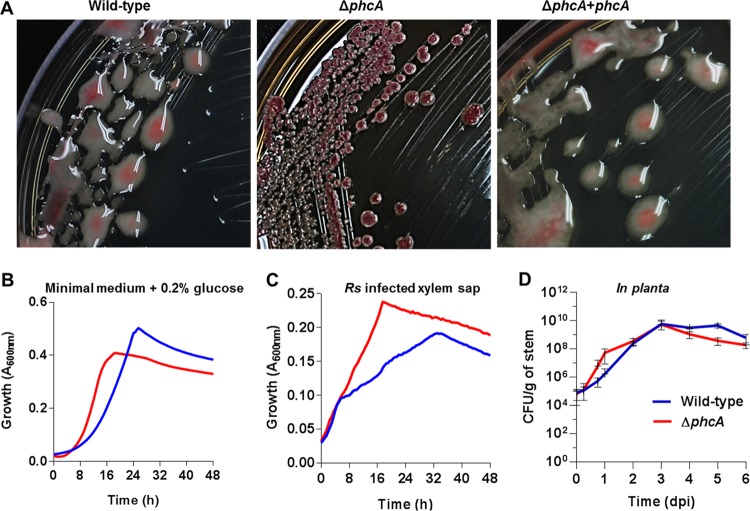
Phenotypes of the low-cell-density-mode-mimicking Δ*phcA* mutant. (A) Colony morphology of *R. solanacearum* strains on tetrazolium chloride agar plates. Wild-type, phylotype I strain GMI1000; Δ*phcA*, deletion mutant of GMI1000; Δ*phcA+phcA*, *phcA* deletion mutant complemented by inserting one copy of *phcA* into the chromosome. (B and C) Growth of wild-type *R. solanacearum* (blue) and of the Δ*phcA* mutant (red) was monitored over 48 h under aerobic conditions at 28°C in minimal broth supplemented with 0.2% glucose (three biological replicates and four technical replicates, *n* = 12) (B) or in *ex vivo* xylem sap harvested from GMI1000-inoculated tomato plants showing early wilt symptoms (four biological replicates, each performed with five technical replicates, *n* = 20) (C). Each replicate was grown in xylem sap pooled from eight plants. Growth was measured as *A*_600_ levels by the use of a BioTek microplate reader. The Δ*phcA* mutant grew faster than the wild type both in minimal medium (Mann-Whitney test, *P* = 0.0081) and in xylem sap (analysis of variance [ANOVA], *P* < 0.0001). (D) Population sizes of the wild-type strain and the Δ*phcA* mutant in tomato stems following inoculation with 2,000 cells through a cut leaf petiole, as determined by serial dilution plating of ground stem slices. Data represent means of results from nine plants per treatment per time point; bars represent standard deviations.

We constructed an *R. solanacearum* strain GMI1000 mutant with a disrupted *phcA* coding sequence to study the behavior of *R. solanacearum* cells that are genetically locked in the low-cell-density mode. As expected, this mutant, here called the Δ*phcA* mutant, formed small round nonmucoid colonies surrounded by brown pigment due to lack of EPS and overproduction of melanin (7) (Fig. 1A). Complementation of the Δ*phcA* mutant by insertion of a copy of the wild-type *phcA* locus and its promoter into the selectively neutral *att* site restored the typical mucoid colony morphology and lack of pigment production (Fig. 1A). The Δ*phcA* strain initially grew faster than the wild type in minimal medium (MM) with glucose (*P* = 0.0081, Mann-Whitney test) (Fig. 1B). However, this high growth rate was not sustained and the wild-type strain ultimately showed a slightly higher maximum level of absorbance. The mutant also grew faster than the wild type in *ex vivo* xylem sap from infected tomato plants, where the Δ*phcA* mutant reached and sustained a higher growth rate than the wild type (Fig. 1C). Together, these data suggest that *R. solanacearum* grows faster in low-cell-density mode than in high-cell-density mode. Finally, we compared the growth kinetics of the two strains in tomato stems following direct inoculation through a cut leaf petiole. Shortly after inoculation, the Δ*phcA* mutant grew faster than the wild-type strain, but the two strains reached similar population levels 3 days postinoculation (dpi) and after day 4, Δ*phcA* growth lagged (Fig. 1D).

### The Δ*phcA* mutant differentially expressed hundreds of genes during tomato colonization.

**FIG 2:**
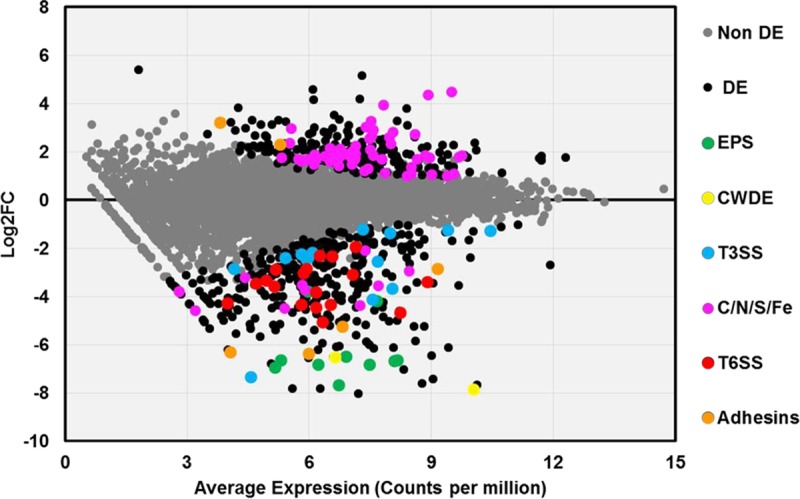
Expression levels of PhcA-responsive genes *in planta*. A scatter plot shows the relationship between the average transcript expression level and log fold change (log_2_FC) for each coding sequence in the *R. solanacearum* genome, based on sequencing of RNA extracted from stems of tomato plants infected with either wild-type or Δ*phcA* bacteria. Log2FC data represent log_2_-transformed values corresponding to reads per kilobase of million mapped reads (RPKM) of the Δ*phcA* mutant versus the wild type. Each circle represents a single coding sequence; black circles indicate genes that are differentially expressed (DE) at a false discovery rate (FDR) of ≤0.005; gray circles indicate non-DE genes. Colored circles indicate DE genes encoding traits of interest: green, extracellular polysaccharide (EPS); yellow, plant cell wall-degrading enzymes (CWDE); blue, structural proteins and effectors of the type III secretion system (T3SS); pink, uptake or catabolism of carbon, nitrogen, sulfur, or iron sources (C/N/S/Fe); red, type VI secretion system (T6SS); orange, hemagglutinin-like proteins and adhesins.

To comprehensively identify traits controlled by the PhcA QS system, and to better understand how this regulator affects pathogen behavior in its natural host environment, we compared the transcriptomes of wild-type and Δ*phcA* strains during colonization of tomato stems. Transcriptome sequencing (RNA-seq) of bacterial RNA harvested from colonized tomato stems 72 h after petiole inoculation, when the population sizes of the two strains were similar, revealed that the PhcA *in planta* regulon is very large. Overall, 620 genes were differentially expressed (DE) in the Δ*phcA* mutant; representing 12.4% of the annotated coding sequences in the GMI1000 genome. Of these, 365 genes were downregulated, and 255 genes were upregulated (Fig. 2). Table S1A in the supplemental material lists all DE genes, defined as those that showed at least a 2-fold change in expression relative to the wild-type strain and that also met the criterion of a stringent false-discovery rate (FDR) of ≤0.005.

10.1128/mBio.00895-17.6 TABLE S1  (A) Complete list of *R. solanacearum* strain GMI1000 genes differentially expressed in a Δ*phcA* mutant relative to the wild type with a 2-fold change at a FDR of ≤0.005. (B) List of putative *R. solanacearum* transporter-encoding genes differentially expressed in the Δ*phcA* mutant *in planta*. (C) List of putative adhesin genes differentially expressed in *R. solanacearum ΔphcA in planta*. Download TABLE S1, PDF file, 0.3 MB. Copyright © 2017 Khokhani et al. 2017 Khokhani et al. This content is distributed under the terms of the Creative Commons Attribution 4.0 International license.

The RNA-seq data were robust and reliable. As expected with RNA extracted from ground plant tissue, most sequence reads mapped to the tomato genome while ~1.2% of the Δ*phcA* reads and ~3.2% of the wild-type reads mapped to the *R. solanacearum* genome (see Fig. S1A in the supplemental material). Results of both multidimensional and scatter plot analyses indicated that the wild-type strain and Δ*phcA* mutant gene expression profiles differed significantly, while those of the biological replicates grouped together (Fig. S1B and C). Hierarchical gene clustering clearly identified many genes that were regulated differently under the two biological conditions (Fig. S1D). Quantitative reverse transcriptase PCR (qRT-PCR) validated the relative expression levels of selected genes of interest; they were consistent with the levels determined by RNA-seq (Fig. S2B).

10.1128/mBio.00895-17.1 FIG S1  Overview of *R. solanacearum* gene expression experiment and grouping of the samples. (A) Mapping summary of RNA-seq data. (B) Multidimensional (MDS) plot constructed using R. The Limma package showed good separation of the results from the wild-type and Δ*phcA* mutant samples. The two axes represent the two dimensions (2-D) of the multidimensional scaling algorithm used to map samples into a 2-D scatter plot based on expression values. (C) Scatter plots showed good correlation among the biological replicates (*r* > 0.9) under the various conditions, whereas the correlation between the wild-type and Δ*phcA* replicates was low (*r* < 0.81). (D) Heat map with hierarchical clustering. On the basis of the standard deviations (SD) of the expression values larger than 30% among the mean expression values (Mean), 916 variable genes were processed by the use of the made4 package from R to cluster genes and samples and to generate a heat map. The color key indicates low (blue) to high (red) normalized expression levels. Gene names are not shown due to the large number of genes used to create the heat map. Download FIG S1, TIF file, 1.9 MB. Copyright © 2017 Khokhani et al. 2017 Khokhani et al. This content is distributed under the terms of the Creative Commons Attribution 4.0 International license.

10.1128/mBio.00895-17.2 FIG S2  (A) Heat map of relative expression levels of virulence factors in wild-type and Δ*phcA R. solanacearum* strains from infected tomato plants. The color key indicates low (blue) to high (red) normalized expression levels in a scale calculated as follows: [log_2_ (average number of reads per kilobase of transcript per million mapped reads) − 5]. (B) qRT-PCR and RNA-seq analyses gave consistent relative *R. solanacearum* gene expression levels. For *in planta* gene expression analyses, susceptible Bonny Best tomato plants were inoculated with the wild-type GMI1000 strain and the Δ*phcA* mutant via the cut petiole method. RNA was isolated from plant stems at the first sign of wilt symptoms. Purified RNA was reverse transcribed into cDNA, and the results were measured by quantitative real-time PCR. The data are presented relative to expression levels in wild-type *R. solanacearum* cells determined under the same conditions. Data shown represent means of results from three biological replicates for each treatment, each of which was performed with two technical replicates (*n* = 6); *rplM* was used as a normalization gene. Gene expression fold changes (log_2_FC) were calculated for each gene in the Δ*phcA* mutant versus the wild-type strain and compared to the log2FC values determined for the same genes from the RNAseq data; bars indicate standard errors of the means. Download FIG S2, TIF file, 0.8 MB. Copyright © 2017 Khokhani et al. 2017 Khokhani et al. This content is distributed under the terms of the Creative Commons Attribution 4.0 International license.

The *in planta* transcriptomic data generally agreed with the previously described phenotypes of *phcA* mutants. As expected, DE genes in the low-cell-density-mimicking mutant included those encoding synthesis of known virulence factors: extracellular polysaccharide (*epsB* and *epsE* [101- and 112-fold downregulated, respectively]), motility and chemotaxis, some type III secretion system structural proteins and several effectors, and plant cell wall-degrading enzymes (endoglucanase *egl* [90-fold downregulated], cellobiohydroloase *cbhA* [228-fold downregulated], and exo-poly-alpha-d-galacturonosidase *pehB* [4.7-fold downregulated]). Expression of the endo-polygalaturonase *pehA* gene, the exo-polygalacturonase *pehC* gene, and the pectin methylesterase *pme* gene, which are downregulated in Δ*phcA* mutants growing in culture (31), trended down but did not meet our criteria for DE *in planta* (Table S1A; Fig. S2A). Along with these known virulence factors, PhcA upregulates the *R. solanacearum* type VI secretion system (T6SS), predicted to translocate factors into either prokaryotic or eukaryotic hosts in a contact-dependent manner (32, 33). This is consistent with the discovery that the cryptic *R. solanacearum* T6SS contributes to virulence (34–36).

### The distinct transcriptome profile of the Δ*phcA* mutant suggests that *R. solanacearum* has a broader catabolic capacity and grows faster in low-cell-density mode.

**FIG 3:**
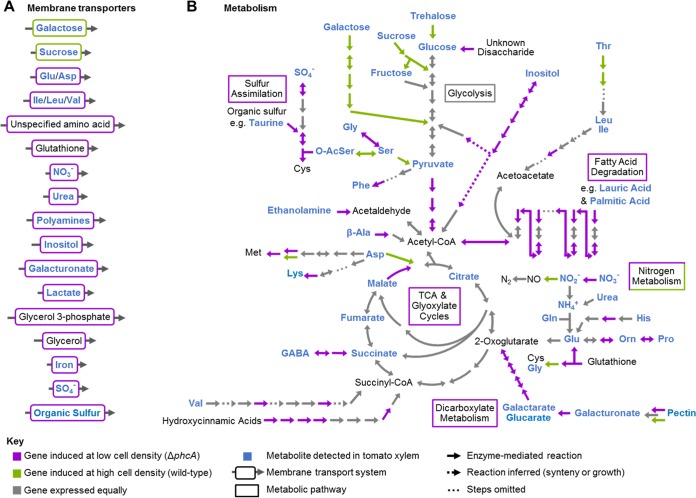
Differentially expressed genes that could enable *R. solanacearum* to use nutrients available in xylem sap. (A) Differentially regulated transporters. See Table S1B for specific gene expression levels. (B) Selected *R. solanacearum* metabolic pathways integrated with transcriptomic results. Metabolic pathways are based on KEGG analysis of the *R. solanacearum* GMI1000 genome. Compounds in blue are present in xylem sap (see references in Table S3). Purple arrows and boxes indicate reaction steps corresponding to enzymes whose expression was upregulated at low cell density (in the Δ*phcA* mutant); green arrows and boxes indicate enzymes whose expression was downregulated at low cell density (in the Δ*phcA* mutant).

Collectively, the annotated functions of the 255 genes upregulated in the Δ*phcA* strain suggested that the pathogen’s core metabolism rates are strikingly different when the bacterium is at low versus high cell densities (Fig. 3; Table S1A). We hypothesized that the low-cell-density-mimicking mutant could grow on sources of carbon, nitrogen, sulfur, and phosphorus that are not available to the pathogen after it reaches high cell density. Around 15% of the upregulated genes in the Δ*phcA* mutant encode putative transporters (Table S1B). These 56 proteins are predicted to transport a wide array of nutrients, including diverse carbohydrates, amino acids, polyamines, iron, nitrate, phosphate, and sulfur. This suggests that *R. solanacearum* avidly scavenges environmental nutrients at low cell densities. Only nine transporter genes were downregulated in the low-cell-density-mimicking mutant.

**FIG 4:**
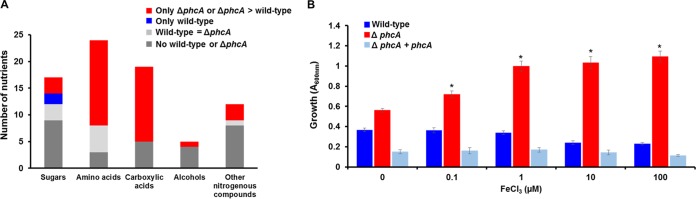
The low-cell-density-mimicking *R. solanacearum* Δ*phcA* mutant uses the nutrients better than the wild-type strain. (A) For each category of available xylem nutrients shown, the stacked bars indicate the number of nutrients that were present in sap from *R. solanacearum*-infected plants that supported growth of the wild-type strain only (blue), the Δ*phcA* mutant only (red), both strains (light gray), or neither strain (dark gray). Bacterial growth was scored by measuring *A*_600_ levels over 72 h at 28°C in minimal broth supplemented with a 10 mM concentration of each indicated nutrient (Table S3) as the sole carbon or nitrogen source. (B) *R. solanacearum ΔphcA* grew better than the wild-type strain under low-iron conditions. *R. solanacearum* strains were grown aerobically in BMM broth supplemented with increasing concentrations of ferric chloride (FeCl_3_). Bacterial growth was measured as absorbance at 600 nm at 24 h. The data show results from three biological replicates, each performed with three technical replicates (*n* = 9). Asterisks indicate differences between the strains (ANOVA, *P* value < 0.001).

To test the hypothesis that low-cell-density-mode *R. solanacearum* cells can access nutrients that are unavailable when the bacterium reaches high cell density, we measured the growth of the wild-type and Δ*phcA* strains on Biolog phenotype arrays and in minimal media supplemented with individual carbon or nitrogen sources. The Δ*phcA* mutant grew better than the wild-type strain on dozens of sugars, carboxylic acids, and amino acids; indeed, the wild-type strain did not grow at all on most of these nutrients (Fig. 4A; Table S2).

10.1128/mBio.00895-17.7 TABLE S2  Growth phenotypes shown by *R. solanacearum* Δ*phcA* on the Biolog phenotype array plates. Download TABLE S2, PDF file, 0.1 MB. Copyright © 2017 Khokhani et al. 2017 Khokhani et al. This content is distributed under the terms of the Creative Commons Attribution 4.0 International license.

10.1128/mBio.00895-17.8 TABLE S3  Biochemistry of the tomato xylem sap habitat occupied by *R. solanacearum* and expression of corresponding catabolic enzyme genes. Download TABLE S3, PDF file, 0.3 MB. Copyright © 2017 Khokhani et al. 2017 Khokhani et al. This content is distributed under the terms of the Creative Commons Attribution 4.0 International license.

The Δ*phcA* mutant upregulated dozens of genes involved in central carbon metabolism (Fig. 3; Table S3). These encode catabolic enzymes such as glucosidase, glycogen synthase, galactaric acid dehydratase, and two glucarate dehydratases. The low-cell-density-mimicking strain also upregulated genes involved in fatty acid metabolism, such as the Swanson conversion of pyruvate to acetyl-coenzyme A (acetyl-CoA), acyl-CoA dehydrogenases and acetyltransferases, and the trifunctional enoyl-CoA hydratase/delta3-cis-delta2-trans-enoyl-CoA isomerase/3-hydroxyacyl-CoA dehydrogenase that catalyzes the β-oxidation of fatty acids (Fig. 3). Conversely, expression of genes encoding degradation of some carbon sources available in xylem sap was repressed in the low-cell-density-mimicking mutant. These include genes encoding endoglucanase, exoglucanase A, trehalase, glycoside hydrolase, and a sucrose-specific phosphoenolpyruvate-carbohydrate phosphotransferase system.

Blast2Go node analysis identified nitrogen metabolism among the biological processes most affected by PhcA (Fig. S4A). Nitrate is relatively abundant in tomato xylem sap, and during pathogenesis *R. solanacearum* strongly expresses several pathways that metabolize inorganic nitrogen (29). Nitrogen assimilation and denitrifying respiration (which enables ATP production in the hypoxic xylem vessels) are both required for normal tomato colonization and bacterial wilt virulence (37, 38). At high cell density, PhcA represses genes encoding nitrate reductase (Table S3) and putative transporters of nitrate, glutamate (*gltJKL*), and glutathione (Table S1B). In contrast, PhcA upregulates nitrite reductase, and direct measurements confirmed that the Δ*phcA* mutant produces more nitrite than the wild type both *in vitro* and *in planta* (Fig. S4B and C). Interestingly, the downstream denitrification steps function whether the quorum sensing system is active or not, because similar levels of nitric oxide were detected in the Δ*phcA* strain, the wild-type strain, and the *phcA*-complemented strain growing under anoxic conditions *in vitro* (Fig. S4D). Several genes encoding the amino acid catabolic pathways that generate tricarboxylic acid (TCA) cycle intermediates (proline, valine, glycine, glutamic acid, or serine) were also DE (Fig. 3; Table S3).

The metabolic capacity of the Δ*phcA* mutant was even broader with respect to sources of phosphorus and sulfur than for carbon and nitrogen sources. The Δ*phcA* mutant grew faster than the wild type on 83% of tested sulfur sources, including sulfate and many other sulfur-containing organic compounds present in soil and xylem sap (39, 40) (Fig. S3B; Table S2). Four sulfur assimilation genes that lead to cysteine synthesis were upregulated in the Δ*phcA* mutant (Fig. 3; Table S3), as well as ABC transporters of sulfate and alkane sulfonate (Table S1B). The low-cell-density-mimicking mutant grew faster than the wild type on 81% of tested sources of phosphorus, which is key to many cellular processes, including signal transduction and energy storage and release. Organic phosphate sources more available to low-cell-density-mode *R. solanacearum* cells included glycolytic intermediates, sugar phosphates, nucleotides, and phospholipid precursors. Together, these results imply that *R. solanacearum* benefits from increased sulfur and phosphorus uptake at low cell densities.

10.1128/mBio.00895-17.3 FIG S3  *In vitro* growth of wild-type and Δ*phcA R. solanacearum* strains. (A) *In vitro* individual inoculations and coinoculations of wild-type and Δ*phcA R. solanacearum*. At 48 h of growth in rich or minimal medium, the cell cultures were serially diluted and plated to obtain CFU counts per milliliter. Each condition was represented by two biological replicates and three technical replicates (*n* = 6). The cell count for the Δ*phcA* strain was different from the count for the wild-type strain under all the tested conditions (*P* value ≤ 0.05 [*t* test]). (B) Biolog phenotypic microarray analysis demonstrating the ability of the wild-type and Δ*phcA* strains to grow on the total number of different types of substrates. For detailed Biolog results, see Table S2. Download FIG S3, TIF file, 0.3 MB. Copyright © 2017 Khokhani et al. 2017 Khokhani et al. This content is distributed under the terms of the Creative Commons Attribution 4.0 International license.

10.1128/mBio.00895-17.4 FIG S4  (A) Pie chart showing the node scores of diverse biological processes based on differentially expressed genes in *R. solanacearum ΔphcA* bacteria growing in tomato stems. Node scores that were calculated using Blast2GO indicate the number of converging annotated sequences of the given gene list at one node; data were also penalized by the distance to the node where each sequence was annotated. Only biological processes with node scores of greater than 27 are shown. Direct and relative measurements of nitrite and nitric oxide levels produced by the respective *R. solanacearum* strains were performed. (B) *In vitro* experiments. Cell populations were started at 5 × 10^6^ CFU/ml; after 18 h under anaerobic conditions, total nitrite levels were measured using the Griess reaction. The data were derived from three biological replicates with three technical replicates each (*n* = 9) (ANOVA, *P* < 0.0001). (C) *In vivo* experiments. The Δ*phcA* mutant accumulated higher nitrite levels than wild-type *R. solanacearum* (ANOVA, *P* < 0.001). Uninfected plants used as controls contained undetectable amounts of nitrite. (D) Nitric oxide (NO) levels produced by *R. solanacearum* strains *in vitro*. NO levels were measured by adding the NO-specific fluorescent dye DAF-FM-DA to VDM broth containing 5 × 10^6^ CFU/ml *R. solanacearum* cells in 96-well microplates. Relative fluorescence levels were measured for 24 h. Data shown here reflect three biological replicates, each containing two technical replicates (*n* = 6). The Δ*norB* mutant, which exhibits hyperaccumulation of NO because it lacks nitric oxide reductase, was included as a positive control. Download FIG S4, TIF file, 0.9 MB. Copyright © 2017 Khokhani et al. 2017 Khokhani et al. This content is distributed under the terms of the Creative Commons Attribution 4.0 International license.

### Low-cell-density-mode-mimicking *R. solanacearum* grew better under low-iron conditions.

Consistent with previous *in vitro* findings (19), we found that genes encoding ferric iron uptake were upregulated in the Δ*phcA* mutant. These included predicted iron-specific transporters and half of the 20 genes related to siderophore synthesis or regulation (Fig. S2A; Table S1A), suggesting that *R. solanacearum* is better able to scavenge iron at low cell densities than at high cell densities. We tested this hypothesis by comparing the growth rates of Δ*phcA* and wild-type bacteria in minimal medium supplemented with different concentrations of ferric chloride. The Δ*phcA* mutant grew better than the wild type under both low-iron conditions and high-iron conditions, but iron concentrations of >1 µM provided no further growth advantage (Fig. 4B). These combined transcriptional and functional results indicate that *R. solanacearum* expends significant resources at low cell densities to obtain iron but that these systems are less active at higher cell densities, possibly because *R. solanacearum* can acquire sufficient iron by other means in the plant.

### The Δ*phcA* mutant is better at using nutrients present in xylem sap than the wild type.

High-cell-density-mode *R. solanacearum* grew more slowly or not at all on many nutrients. Does PhcA repress catabolism of these nutrients because they are not present in xylem sap? To characterize the nutrients available to *R. solanacearum* during tomato pathogenesis, we compiled a list of compounds known to be present in tomato xylem sap, based on published analyses of sap from healthy plants and on our untargeted metabolomic analysis of sap from *R. solanacearum*-infected wilt-susceptible tomato cultivar “Bonny Best” (Table S3) (29, 38, 41–44). Transcriptomic data suggested that many of the metabolites present in tomato xylem sap (highlighted in blue in Fig. 3) are more accessible to the low-cell-density-mimicking Δ*phcA* mutant. We functionally validated the RNA-seq results by comparing the growth rates of the wild-type and Δ*phcA* strains on selected compounds from this list used either as the sole carbon source or, for N-containing compounds, as the sole nitrogen source (Table S3). The Δ*phcA* mutant was better able to use 36 of 76 tested xylem sap nutrients (Fig. 4A; Table S3). Nine metabolites were used similarly by the two strains, and only galactose and trehalose supported growth of the wild-type strain alone. The remaining 29 tested xylem sap compounds did not support growth of either strain under our conditions. Thus, *R. solanacearum* can grow faster on many nutrients present in tomato xylem sap at low cell densities than it can after reaching high cell density. This may facilitate rapid pathogen growth at the earliest stages of host colonization.

However, the Δ*phcA* mutant also grew better than the wild type on 18 carbon or nitrogen sources that are not known to be present in xylem sap, such as butyric acid, tartaric acid, and cysteine (Table S2). Upregulating the corresponding transporters and catabolic pathways at low cell densities could increase the fitness of *R. solanacearum* outside plants by enabling the bacterium to access diverse nutrients present in low concentrations in soil and water.

### PhcA affects *R. solanacearum* attachment to abiotic surfaces.

**FIG 5:**
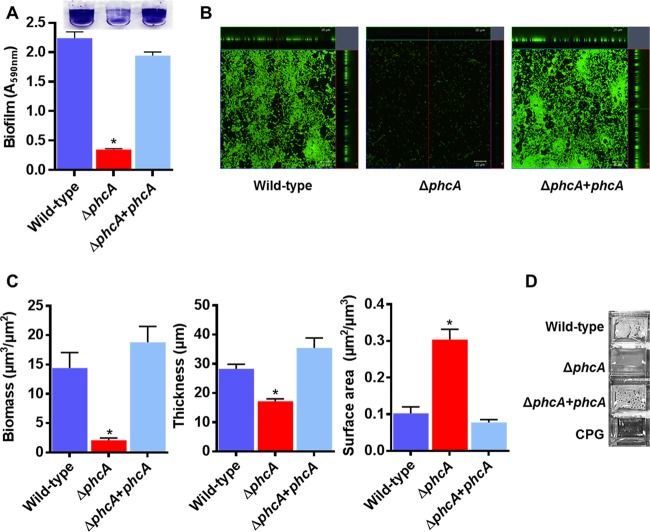
PhcA is required for normal *R. solanacearum* attachment to abiotic surfaces. (A) Biofilm formation by *R. solanacearum* strains was quantified using the PVC plate-crystal violet staining method; data shown reflect results from two biological replicates, each performed with 12 technical replicates (*n* = 24); the asterisk indicates *P* < 0.0001 (ANOVA). (B) Confocal laser microscopy images showing orthogonal views of biofilm formed by *R. solanacearum* cultures after 24 h of static incubation in a glass chamber slide and staining performed with a BacLight Live/Dead kit. Live cells are green, and dead cells are red. Images are representative of results from two biological replicates with four technical replicates each (scale bar in inset, 20 µm). (C) *R. solanacearum* Δ*phcA* mutant cells produce *in vitro* biofilms on glass that are thinner and more diffuse than those of wild-type cells. Comstat analysis was used to calculate the biomass, thickness, and surface area of 24-h biofilms (*n* = 8). Asterisks indicate *P* < 0.05 (ANOVA). (D) Pellicles formed by *R. solanacearum* strains on the surface of rich broth after 48 h of static incubation; images are representative of results from three biological replicates, each performed with two technical replicates.

*R. solanacearum* forms biofilm-like aggregates inside xylem vessels in wilting plants, and *R. solanacearum* mutants that make abnormally thick biofilms *in vitro* are reduced in virulence (45, 46). Interestingly, many genes potentially associated with bacterial attachment, including six that encode hemagglutinin-like adhesin proteins, were differentially expressed in the low-cell-density-mimicking Δ*phcA* mutant. Three hemagglutinin genes were strongly upregulated, while three others were downregulated, suggesting that the PhcA QS system modifies the ability of *R. solanacearum* cells to aggregate and/or to adhere to surfaces in complex ways (Fig. S2A; Table S1C). To better understand this trait, we measured bacterial attachment to abiotic surfaces *in vitro*. The Δ*phcA* mutant formed much less biofilm than the wild type on polyvinyl chloride (PVC) plates stained with crystal violet (Fig. 5A). Confocal imaging analysis of 24 h biofilms on glass plates revealed that the Δ*phcA* mutant did not form structured three-dimensional biofilms like wild-type *R. solanacearum*, possibly because it was arrested in a low-cell-density mode optimized for initial substrate attachment. The Δ*phcA* mutant attached to a 3-fold-larger area than the wild-type strain or complemented mutant strain (*P* < 0.01, *t* test) (Fig. 5B and C). The Δ*phcA* biofilms were 2-fold thinner and contained 7-fold less biomass than those of the wild type (*P* < 0.05, *t* test). After 48 h of static growth in rich medium, the wild-type cells formed a floating pellicle at the air-liquid interface, but the Δ*phcA* mutant did not (Fig. 5D). Although this distinctive phenotype suggested that low-cell-density-mode *R. solanacearum* cells do not self-aggregate as well as they do at high cell density, its biological significance in a natural context remains to be determined.

### Δ*phcA* cells colonizing tomato xylem are morphologically different from wild-type cells.

**FIG 6:**
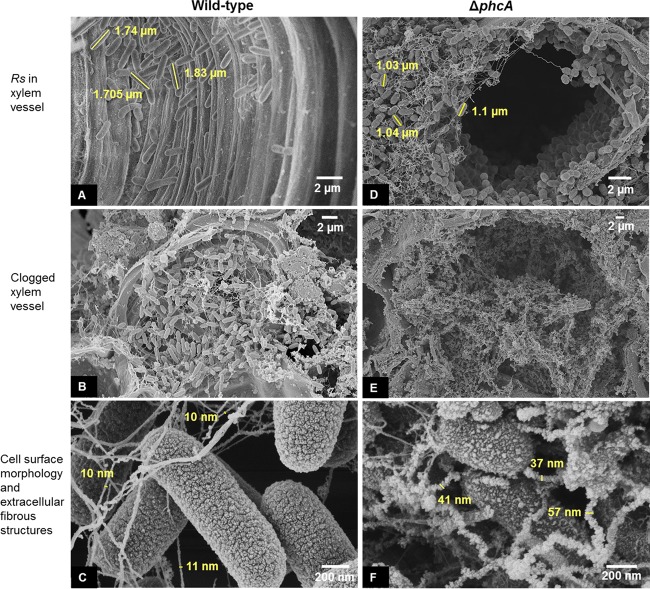
Δ*phcA* cells colonizing tomato xylem differ qualitatively from wild-type cells. Representative SEM images show stem cross sections of tomato plants infected by the *R. solanacearum* (*Rs*) wild-type strain (A to C) and the Δ*phcA* mutant (D to F) 72 h after cut-petiole inoculation. For each treatment, SEM images were captured from two biological replicates, each performed with six technical replicates.

We used scanning electron microscopy (SEM) to image colonized tomato stem tissue harvested at 72 h postinoculation, when the wild-type and Δ*phcA R. solanacearum* cells had reached comparable population sizes. This is the same time point at which their transcriptomes were profiled. The wild-type bacteria had colonized most of the xylem vessels examined, where they formed loose aggregates of cells connected by thin, smooth fibers (Fig. 6B). In contrast, Δ*phcA* cells were present in fewer xylem vessels. In the vessels that were colonized, the Δ*phcA* bacteria formed dense mats of cells embedded in fibers that were more numerous, more kinked, and rougher than the fibers in the wild-type aggregates (Fig. 6D, E, and F). These fibers were on average four times thicker than those in the wild-type cell aggregates. *In planta*, the Δ*phcA* cells were on average 25% shorter in their longest dimension than the wild-type cells (Fig. 6A and D). The Δ*phcA* cells were oval, in contrast to the rod-shaped wild-type cells (Fig. 6A and D). Finally, the surfaces of the Δ*phcA* and wild-type cells had different textures in the SEM images; the wild-type cells had a uniform, rugose surface whereas the Δ*phcA* cell surfaces appeared more irregular, possibly reflecting the absence of EPS (Fig. 6C and F). Overall, the wild-type and Δ*phcA* bacteria were qualitatively different during tomato plant colonization with respect to their distribution; aggregate matrix composition; and the size, shape, and surface texture of their cells.

### The hyperattachment behavior of the Δ*phcA* mutant restricts dispersal in the host.

**FIG 7:**
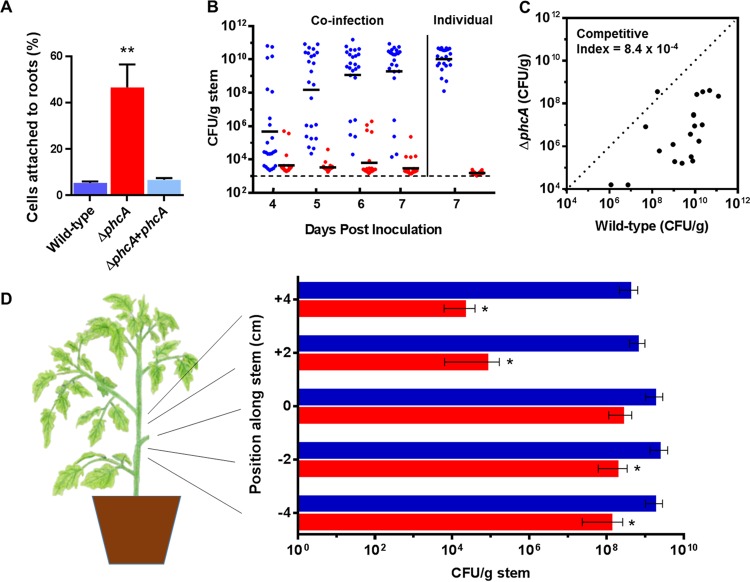
The low-cell-density-mode-mimicking *R. solanacearum ΔphcA* mutant hyperattached to tomato roots but had reduced competitive fitness *in planta* and was defective in plant colonization. (A) Root attachment. Four-day-old tomato seedlings were incubated for 2 h in water with wild-type, Δ*phcA* mutant, or complemented Δ*phcA* mutant cells and were then rinsed, ground, and dilution plated to quantify attached bacteria. Results reflect three biological replicates, each performed with 10 technical replicates (*n* = 30). The asterisk indicates that the Δ*phcA* mutant strain attached better to roots than the wild-type and complemented mutant strains (*P* < 0.0001, ANOVA). (B) Competitive fitness of wild-type versus Δ*phcA* bacteria following soil-soak inoculation. Unwounded tomato plants were coinoculated by soaking soil with 1:1 suspensions of reciprocally marked wild-type and Δ*phcA* bacteria. Tomato mid-stems were sampled over time, and the population size of each strain was determined by serial dilution plating on relevant selective media. For comparison, bacterial population sizes in plants inoculated with only one strain are shown on the right. The wild-type strain outcompeted the Δ*phcA* mutant at all time points (*P* < 0.0001 [Wilcoxon signed-rank test; *n* = 12 plants per inoculation per time point]). (C) Competitive fitness of Δ*phcA* and wild-type bacteria in tomato stems following direct stem inoculation. Tomato plants were coinoculated via a cut leaf petiole with 2,000 CFU of reciprocally marked wild-type and Δ*phcA* bacteria in a 1:1 suspension. At 72 hpi, the population size of each strain 4 cm above the inoculation site was determined by dilution plating. The median competitive index value determined for the Δ*phcA* mutant was 8.4× 10^−4^ (*P* < 0.0001 [Wilcoxon signed-rank test; *n* = 10 plants per coinoculation, 20 total]). (D) Dispersal of wild-type and Δ*phcA* strains in tomato stems. Petioles of 3-week-old tomato plants were inoculated with 2,000 cells of either wild-type or Δ*phcA* bacteria. After 72 h, stem sections were harvested at 0, 2, and 4 cm above and below the point of inoculation, and bacterial population sizes were determined by dilution plating. Asterisks indicate *P* < 0.01 (Wilcoxon signed-rank test; *n* = 8 plants per treatment). Bars represent standard errors of the means.

Because SEM analysis revealed that the Δ*phcA* mutant formed denser aggregates in xylem vessels than the wild type, we predicted that the Δ*phcA* mutant would adhere more avidly to plant surfaces. We compared the levels of attachment of wild-type and Δ*phcA* bacteria to tomato seedling roots. Nine times more Δ*phcA* cells than wild-type cells were attached to the roots after 2 h (Fig. 7A). This is consistent with the idea that at low cell density, *R. solanacearum* cells adhere better to plants, possibly as a result of higher expression of adhesins specific to biotic surfaces. Together, the increased root attachment and limited xylem colonization by the Δ*phcA* mutant suggested that low-cell-density-mimicking cells might not disperse well inside host plants. However, our metabolic analyses and the observation that Δ*phcA* cells grew faster than wild-type cells in artificial media (Fig. S3A) and in *ex vivo* xylem sap (Fig. 1C) suggested that cells mimicking the low-cell-density condition would grow faster *in planta* and outcompete the wild-type strain.

To test these opposing hypotheses, we compared the abilities of the Δ*phcA* mutant to colonize tomato plants alone and in the presence of wild-type cells. When tomato plants were infected with a 1:1 suspension of wild-type and Δ*phcA* cells using a naturalistic soil-soak inoculation, the wild-type strain consistently outcompeted the Δ*phcA* mutant in tomato mid-stems (Fig. 7B). Even at 7 days after inoculation, few Δ*phcA* cells were detected in stems of coinoculated plants, although most plants contained more than 10^8^ CFU of the wild-type strain per g stem at that time point (Fig. 7B). Interestingly, this defect was even more pronounced when the Δ*phcA* mutant was inoculated alone, suggesting that wild-type cells partially complement the Δ*phcA* colonization defect in *trans*, possibly by producing EPS or other secreted virulence factors (Fig. 7B). Similar results were obtained following direct coinoculation into the stem through a cut leaf petiole (Fig. 7C). Kinetic analysis showed that in the first 2 days after coinoculation, the Δ*phcA* mutant grew better than the wild type at the site of inoculation, possibly due to its ability to better exploit the nutrients available in tomato xylem (Fig. S5). However, the wild-type cells quickly caught up, and by 5 dpi the wild-type bacteria outnumbered the Δ*phcA* cells (Fig. 7C and S5). Moreover, following coinoculation, Δ*phcA* cells were defective in moving up tomato stems, with a very poor competitive index (CI) of 8.4 × 10^−4^ at 4 cm above the site of inoculation (Fig. 7C).

10.1128/mBio.00895-17.5 FIG S5  *In planta* kinetics of competitive growth levels of the wild-type and Δ*phcA* strains in tomato following petiole inoculation. Petioles of 3-week-old tomato plants were inoculated with a 1:1 ratio of wild-type and Δ*phcA* cells. At different time points postinfection, a thin section of stem tissue from the point of inoculation was harvested, ground, and dilution plated to determine the cell density of *R. solanacearum* (*P* < 0.05 [Wilcoxon signed-rank test, *n* = 9]). Error bars represent standard deviations. Download FIG S5, TIF file, 0.1 MB. Copyright © 2017 Khokhani et al. 2017 Khokhani et al. This content is distributed under the terms of the Creative Commons Attribution 4.0 International license.

To determine if the low-cell-density-mode-mimicking Δ*phcA* strain could spread as well as the wild-type strain in tomato stems, we measured the population sizes of each strain above and below the inoculated petiole 7 days after inoculation. The Δ*phcA* mutant was strikingly deficient in the ability to move both up and down tomato stems (Fig. 7D). At 4 cm above the inoculation point, the mean population size of the wild-type strain was 4 × 10^8^ CFU/g stem compared to only 2 × 10^4^ CFU/g stem for the Δ*phcA* mutant.

Overall, the behavior of the Δ*phcA* mutant *in planta* suggests that it is adaptive for *R. solanacearum* to adhere to plant surfaces and to form aggregates there at early stages of infection. However, as the bacterium grows to high population densities in xylem vessels, this hyperattachment trait must be repressed so that the pathogen can disperse in the host plant.

### The Δ*phcA* mutant elicited lower tomato defense gene expression than the wild type.

Infection with *R. solanacearum* causes tomato plants to increase expression of genes in both the ethylene (ET) and salicylic acid (SA) defense pathways (47). To determine if low-cell-density-mode-mimicking cells triggered plant defense gene expression as well as the wild type, we performed quantitative RT-PCR analysis of total RNA harvested from tomato stems 72 h postinoculation (hpi) with either Δ*phcA* or wild-type bacteria. Plants infected with comparable populations of the Δ*phcA* mutant expressed about 3-fold less of the SA-responsive defense gene *PR-1a* (*P* = 0.007, *t* test) and about 4-fold less of the ET-responsive defense gene *ACO5* (*P* = 0.033, *t* test) than plants infected with the wild-type strain. Expression of the jasmonic acid (JA) pathway marker *Pin2* was not altered (*P* = 0.64, *t* test).

## DISCUSSION

To understand how *R. solanacearum* makes the critical transition from living as scattered cells in water, soil, or on root surfaces to the very different conditions it experiences at high population densities in plant xylem vessels, we needed to profile the pathogen’s gene expression at low cell density. Harvesting quality RNA from sparse, scattered cells is technically challenging, so we constructed a QS-blind Δ*phcA* mutant that mimics the behavior of the pathogen at low population densities. The transcriptomes of the Δ*phcA* mutant and wild-type *R. solanacearum* indirectly defined the metabolic and behavioral traits that help *R. solanacearum* succeed at low cell densities.

The PhcA quorum sensing system enables *R. solanacearum* to use different nutrient sources at low and high cell densities. Combining transcriptomic data with functional assays and xylem sap metabolomics identified specific mechanisms that expand the carbon sources available to *R. solanacearum* under low-nutrient conditions. Many metabolic pathways and transporters were expressed only at low cell densities, and functional assays confirmed that high-density *R. solanacearum* could not grow on the corresponding carbon sources. Thus, the pathogen can access some nutrients only at low cell densities and can also grow faster on many nutrients that are accessible to both modes. This suggests that the PhcA QS system regulates *R. solanacearum* core metabolism in ways that maximize growth early in colonization but that slow growth once bacteria reach high cell densities.

Among 76 potential nutrient sources detected in tomato xylem, wild-type *R. solanacearum* (high-cell-density mode) grew better than the Δ*phcA* mutant only on trehalose and galactose, while the Δ*phcA* mutant grew better or exclusively on 47% of the tested xylem metabolites. Several compounds that feed directly into central carbon metabolism (glycolysis and the TCA cycle) are more available to low-cell-density-mode-mimicking *R. solanacearum* cells. These include pyruvic acid, galactaric acid, glucaric acid, malate, citrate, and several others. Malate and citrate are important for the success of other plant-pathogenic bacteria such as *Pseudomonas syringae* and *Xanthomonas campestris* pv. *vesicatoria* (48, 49).

*R. solanacearum* colonizes dead and lignified xylem tissue, which contains diverse carbohydrates such as glycosides, glycans, and glycoconjugates (50). Glucosidases convert these complex sugars to glucose, which is a preferred carbon source for *R. solanacearum* at both high and low cell densities. Glucosidase expression is upregulated 7-fold in Δ*phcA* cells, suggesting that host cell wall fragments are important carbon sources early in plant colonization. Conversely, trehalose and galactose, which are accessible only to high-cell-density-mode *R. solanacearum*, are likely key nutrients during full-blown wilt disease. Strategic use of a preferred carbon source such as glucose when the bacterium is at low cell densities not only provides the fitness advantage of rapid growth early in xylem colonization but also allows *R. solanacearum* to exploit less-preferred nutrient sources when it has already reached the critical density required for virulence. Further, rapid uptake of glucose, a widely preferred microbial carbon source, may help *R. solanacearum* outcompete other rhizosphere and endophytic bacteria.

Genes encoding nitrogen metabolism were upregulated in the low-cell-density-mimicking mutant, and the Δ*phcA* mutant grew better than the wild type on many nitrogen sources, including nitrate, nitrite, ammonia, and 16 amino acids. Plants infected with the Δ*phcA* mutant contained higher levels of nitrite, which *R. solanacearum* can assimilate and convert into glutamine and glutamate via ammonia (51). These amino acids are present in tomato xylem sap, and the GltIJKL glutamate transporters and glutamate dehydrogenase GdhA were upregulated in the Δ*phcA* mutant, consistent with increased assimilation of these compounds at low cell densities. In addition to representing acquisition of nitrogen for growth, increased nitrite levels may improve *R. solanacearum* survival during initial infection in the hypoxic host environment (52). Nitrates protect *Mycobacterium tuberculosis* from hypoxic stress by replacing oxygen as the terminal electron acceptor in the respiratory chain (53). The *M. tuberculosis* nitrate importer NarK2 is highly induced during hypoxia to levels similar to those that *R. solanacearum* encounters in tomato xylem (38). The upregulation of NarK2 in the Δ*phcA* mutant, along with the finding that denitrifying respiration is critical for *R. solanacearum* success during pathogenesis (38), suggests that nitrate may play a similar protective role for *R. solanacearum in planta*.

A recent *in silico* model of *R. solanacearum* strain GMI1000 metabolism also suggested that a *phcA* mutant had a generally broader catabolic capacity than the wild type, with specific functional results mostly consistent with our data (54). However, in contrast to their report, but in agreement with our transcriptomic data, we found that the Δ*phcA* mutant did not grow on galactose either in the Biolog galactose or in minimal medium (MM) plus galactose. This difference may have been a consequence of the fact that Peyraud and coworkers starved *R. solanacearum* cells in water for 6 h before inoculating the Biolog plates, whereas we harvested metabolically active bacteria from mid-log phase. Minimal medium and Biolog growth assays gave inconsistent results for a few other key carbon sources, such as sucrose, trehalose, and glycerol.

Downregulation of genes encoding a trait did not always lead to loss of function. For example, despite the low expression levels of *scrAB* sucrose transport and catabolism genes in the Δ*phcA* mutant, the low-cell-density-mimicking mutant grew as well as the wild type in MM supplemented with 10 mM sucrose. Expression of the *scrRAB* locus is induced by sucrose; if the proprietary Biolog medium contains little sucrose, this could explain why the mutant did not grow in the Biolog assay (29, 55). This carbon source is important in bacterial wilt disease development; a *scrA* mutant of *R. solanacearum* strain UW551 is reduced in virulence in tomato (29).

Taken together, the results of our transcriptomic and functional assays demonstrate that *R. solanacearum* uses many sources of carbon, nitrogen, sulfur, phosphorus, and iron more efficiently at low cell densities than when it is in high-cell-density mode. Indeed, the pathogen can use many of these substrates only at low cell density. How does this broad metabolic shift increase pathogen fitness? Perhaps the low-cell-density-mode bacteria are optimized to use nutrients available outside the plant in more chemically diverse and nutrient-poor rhizospheres and surface water habitats (56). This environmental nutrition hypothesis is consistent with the ability of the Δ*phcA* mutant to use compounds that have not been reported to be present in tomato xylem sap, including sulfur sources such as cysteine and glutathione. These may contribute to bacterial success in nonnutritional ways. For example, *R. solanacearum* at low cell densities could protect itself from oxidative damage inside the host plant by accumulating glutathione via upregulation of the *gsiABCD* glutathione ABC transporter and the *ggtI* and *gstH* glutathione S-transferases (57, 58).

Surprisingly, quorum sensing does not optimize high-cell-density-mode *R. solanacearum* to use nutrients in tomato xylem sap, where the bacterium multiplies rapidly (59). Instead, PhcA represses many catabolic pathways so that, once it exceeds 10^7^ CFU/ml, *R. solanacearum* uses only a few of the nutrients that it encounters during pathogenesis. We favor the hypothesis that low-cell-density-mode *R. solanacearum* more efficiently exploits compounds in xylem sap to fuel the pathogen’s explosive growth early in disease development, before the QS system is activated. But the pathogen no longer uses all available xylem nutrients once it reaches high cell density and begins to express virulence factors. These findings are consistent with an *in silico* metabolic model suggesting that the PhcA QS system mediates a trade-off between optimal growth early in plant colonization and resource-demanding production of virulence factors later in disease development (54).

Similar trade-offs have been identified in many other bacteria (60). Environmental constraints and finite resources force bacteria to choose between two demanding traits at different developmental or disease stages (89). Their broader catabolic capacity and more rapid growth explain why spontaneous *phcA* mutants grow faster than the wild type in culture (7). Peyraud et al. proposed that spontaneous *phcA* mutants, which occur frequently (6), should outcompete wild-type cells in plants (54). We tested this hypothesis and found that it is not the case: wild-type cells outcompeted the Δ*phcA* mutant *in planta* by more than 10,000-fold. Moreover, *phcA*-type cells are rarely or never isolated from infected plants in the field (7). This suggests that, inside the plant, the growth advantage of *phcA*-type cells is outweighed by other disadvantageous traits. What are these traits?

The bacterium’s ability to adhere to biotic and abiotic surfaces is dramatically modified by *R. solanacearum* cell density. While Δ*phcA* cells did not form biofilms on abiotic substrates, they attached to roots much better than wild-type cells. Avidly binding to roots of potential hosts would be adaptive for *R. solanacearum* at low cell densities in soil. However, SEM images revealed that once they were inside a plant, Δ*phcA* cells formed abnormally thick, matted aggregates. All these phenotypes may be the result of altered expression of hemagglutinin-related nonfimbrial adhesins in the Δ*phcA* mutant. Adhesins are surface proteins that help bacteria attach to eukaryotes, an essential step in host-microbe interactions (61). Nonfimbrial adhesins influence the attachment behavior of phytopathogenic bacteria such as *Xylella fastidiosa* and *Xanthomonas fuscans*, which are initially bound to a surface by nonfimbrial adhesins (62–65). Once bacteria attach to a surface, fimbrial adhesins (pili, fimbriae) help bacteria stick to each other and form aggregates on the host surface. In general, adhesins contribute to virulence by playing specific roles at different disease stages (early infection, seed transmission, attachment to insect vectors, etc.). Nutrient availability can also influence attachment behavior; under low-nutrient conditions, *Acinetobacter* cells attached with increased stability and formed less-dispersive biofilm structures (66).

The *R. solanacearum* genome encodes about 20 putative hemagglutinin-related proteins, of which 6 were regulated by PhcA; 3 of the 6 showed higher expression at low cell densities, and the other 3 showed higher expression at high cell densities. A systematic analysis of mutants lacking these differentially expressed adhesins would identify their roles in attachment to various substrates throughout the disease cycle.

The low-cell-density-mimicking mutant upregulated genes encoding degradation of hydroxycinnamic acids (HCAs). These plant-produced compounds inhibit *R. solanacearum*, and we previously found that HCA degradation protects *R. solanacearum* from HCA toxicity in tomato roots, where these compounds are abundant (67). This is consistent with the hypothesis that, at low cell densities, the PhcA QS system upregulates traits that the pathogen needs early in disease development.

Inside the plant, the morphology of Δ*phcA* cells was strikingly different from that of wild-type cells: the Δ*phcA* cells were smaller, rougher, and more oval. Similar morphological differentiation was seen in stationary-phase *Escherichia coli* and *Arthrobacter* cells undergoing a survival response (68, 69). Under low-nutrient conditions, *Acinetobacter* sp. strain GJ12 cells switch from bacillar to coccoid morphology (66), which increases their surface-to-volume ratio and accommodates more nutrient transporter complexes (70). The increased roughness of the Δ*phcA* cell surfaces may be due to lack of EPS (71). Adding exogenous EPS to a spontaneous EPS-deficient rough mutant of *R. solanacearum* facilitated escape from agglutination (71), consistent with a role for EPS in colonization.

The Δ*phcA* cells were defective in plant colonization, occupying fewer xylem vessels and spreading up and down stems more slowly than the wild-type cells, possibly because they could not escape their abnormally matted aggregates. QS mutants of *Pantoea stewartii* subsp. *stewartii* also remained localized at the site of infection (72). Conversely, an *rpfF* QS mutant of *Xylella fastidiosa* had reduced adhesiveness, spread better in grapevine vessels than the wild type, and was hypervirulent after mechanical inoculation (73). Nonetheless, that mutant had very low fitness overall because it did not form biofilms in its insect vector and was poorly transmitted (74). *R. solanacearum* biofilms contain fibers made of extracellular DNA, and endonuclease-deficient mutants produced thick biofilms and spread poorly in tomato (46). A putative nuclease-encoding gene, RSp1603, was downregulated 35-fold in the Δ*phcA* mutant. This may preserve the structural integrity of developing biofilms by protecting the DNA fibers. Further investigation is needed to explore the connection between extracellular DNA degradation and the structure of Δ*phcA* aggregates *in planta*.

The hyperattachment trait of the Δ*phcA* mutant, which is consistent with its reduced competitive fitness and poor dispersal in plant stems, suggests that despite having the advantage of broad metabolic capacity, spontaneous *phcA* mutants are at a strong selective disadvantage *in planta*. Their altered morphology, root attachment, and aggregation suggest that these behaviors are advantageous at low cell densities in soil and early in plant colonization but must be downregulated for the pathogen to successfully colonize and spread as disease develops.

In an interesting parallel, it is well established that plants also execute an evolutionary trade-off between maximizing growth and maximizing defenses (75). Plants responded differently to *R. solanacearum* cells in the low-cell-density and high-cell-density modes. Wild-type *R. solanacearum* infection triggers rapid induction of salicylic acid and ethylene-responsive defense pathway genes, but both of these host signaling pathways were significantly less induced by the Δ*phcA* mutant, even when comparable numbers of pathogen cells were present. Similarly, human bronchial epithelial cells produced much less of the defensive cytokine interleukin-8 in response to a QS mutant of *Pseudomonas aeruginosa* than in response to wild-type *P. aeruginosa* (76).

The *R. solanacearum* QS mutant is compromised in expression of multiple virulence factors, some of which may trigger plant recognition and defense. It would clearly be advantageous for *R. solanacearum* to avoid activating host defenses early in plant colonization. Thus, reduced expression of these virulence factors not only saves energy but may also make *R. solanacearum* less visible to plant defenses. The lack of EPS in the Δ*phcA* mutant cannot explain this reduced defense gene expression because an EPS-deficient *R. solanacearum* mutant still induces wild-type defenses in wilt-susceptible plants such as the cultivar used here (47). Some *R. solanacearum* T3-secreted effectors suppress host defenses, but many T3SS-encoding genes were downregulated in the Δ*phcA* mutant, including those encoding the system’s major transcriptional activators, HrpB and HrpG. Nonetheless, the low-cell-density-mimicking Δ*phcA* mutant ultimately failed to colonize plants. It is possible that the mutant’s inability to spread normally meant that, overall, fewer plant cells perceived the pathogen, leading to a muted defense response. This could be tested using laser-capture microdissection to measure defenses in single plant cells that are in contact with either wild-type or Δ*phcA* bacteria.

Along with identifying new QS-regulated phenotypes, this study also revealed some inconsistencies between the *R. solanacearum* gene expression levels measured in culture and the pathogen’s transcriptional behavior in the natural environment of the host plant (summarized in Table S5). Most notably, the expression of the major T3SS regulator HrpB in culture is repressed by PhcA at high cell densities; this generated a model proposing that T3 secreted effectors were important only early in plant colonization (27). However, we found that many T3SS-related genes, including *hrpB*, were actually more highly expressed at high cell densities *in planta*. This is consistent with previous *in planta* experiments showing that T3SS genes are highly expressed during disease (28–30). It will be interesting to identify the intervening regulatory players that reverse this regulatory pathway in the plant.

10.1128/mBio.00895-17.9 TABLE S4  Strains, plasmids, and primers used in this study. Download TABLE S4, PDF file, 0.3 MB. Copyright © 2017 Khokhani et al. 2017 Khokhani et al. This content is distributed under the terms of the Creative Commons Attribution 4.0 International license.

10.1128/mBio.00895-17.10 TABLE S5  Comparison of *R. solanacearum* trait regulation in culture and *in planta*. Download TABLE S5, PDF file, 0.2 MB. Copyright © 2017 Khokhani et al. 2017 Khokhani et al. This content is distributed under the terms of the Creative Commons Attribution 4.0 International license.

Importantly, the gene expression levels reported here reflect averages across biologically heterogeneous bacterial populations that occupy distinct microenvironments. We extracted *R. solanacearum* RNA from infected tomato stem tissue, which included both the planktonic bacteria and those in aggregates or attached to plant surfaces. It is very likely that early in disease, cells in biofilms are in a different QS mode than individual cells in flowing xylem sap. Follow-up experiments could distinguish between gene expression levels in planktonic versus attached cells in xylem by using reporter gene fusions to compare levels of transcription of PhcA-responsive genes across subpopulations (77).

In conclusion, the *in planta* PhcA transcriptome identified novel traits that help *R. solanacearum* survive at low cell densities. The PhcA QS system controls two transitions essential for successful bacterial wilt pathogenesis. First, *R. solanacearum* expresses diverse transporters and catabolic pathways to maximize survival in soil or water and growth during early infection, but once infection has been established, these traits are repressed by PhcA in favor of virulence factor expression. Second, PhcA modulates the switch between a highly adhesive form that forms aggregates and attaches avidly to host surfaces and a more dispersive form that spreads rapidly within plants during host colonization. Because these transitions clearly play critical roles in the pathogen’s complex disease cycle, they are attractive targets for disease control. Exogenously applied, microbiome-delivered, or host plant-produced QS inhibitors, quorum quenchers, or QS autoinducers (78, 79) could effectively reduce *R. solanacearum* success and, by extension, reduce crop losses due to bacterial wilt disease.

## MATERIALS AND METHODS

### Bacterial strains and growth conditions.

Bacterial strains used in this study are listed in Table S4 in the supplemental material. *Escherichia coli* cells were grown at 37°C in LB medium. *R. solanacearum* was grown at 28°C in Boucher’s minimal medium (BMM) broth (pH 7.0), Casamino Acids-peptone-glucose (CPG) rich broth, or Van den Mooter (VDM) denitrification broth; in *ex vivo* tomato xylem sap; or on tetrazolium chloride (TZC) agar plates (80, 81). Media were supplemented with gentamicin (Gm) (15 µg/ml) or tetracycline (Tc) (5 µg/ml) as necessary. Bacterial growth was assessed in BMM broth supplemented with various concentrations of ferric chloride (FeCl_3_) or with 10 mM carbon or nitrogen sources, using a microplate reader (Bio-Tek, Winooski, VT) with shaking at 28°C under aerobic conditions. Biolog PM1-4 phenotype array plates were used according to the instructions of the manufacturer (Biolog Inc., Hayward, CA). Kinetic data were analyzed at 72 h using Omnilog software. Growth in *ex vivo* xylem sap was measured as described previously (67), using three biological replicates each with at least three technical replicates. To measure nitric oxide levels, cells were grown in VDM medium supplemented with 4-amino-5-methylamino-2′,7′-difluorofluorescein diacetate (DAF-FM-DA) and relative fluorescence was measured by the use of a plate reader. The Δ*norB* strain of *R. solanacearum*, which accumulates nitric oxide (38), was used as a positive control.

### Plant assays.

Plant assays were conducted on wilt-susceptible Bonny Best tomato plants inoculated through unwounded roots by soil drenching or directly into stems through a cut leaf petiole as previously described (67). To compare the levels of *in planta* spread of the wild-type and Δ*phcA* strains, bacteria were enumerated by dilution plating of ground stem slices harvested 0, 2, and 4 cm above and below the point of inoculation (46).

To assess the *in planta* competitive fitness of the wild-type and Δ*phcA* strains, plants were soil-soak inoculated with a 1:1 ratio of wild-type and Δ*phcA* cells totaling 10^8^ CFU/ml and bacterial populations in mid-stems were enumerated as described above at 4, 5, 6, and 7 days after inoculation. Population sizes were normalized to the initial inoculum of each strain. The experiment included two biological replicates, each performed with 12 plants per condition (*n* = 24). To measure bacterial competitive fitness in tomato stems, a 2-μl drop of bacterial suspension containing 2,000 cells in a 1:1 mixture of marked wild-type and Δ*phcA* bacteria was placed on a freshly cut leaf petiole. After 72 h, strain population sizes were measured in stems 4 cm above the point of inoculation by serial dilution plating in duplicate on antibiotic-containing media specific for each strain. The experiment included two biological replicates, each performed with 10 plants per condition (*n* = 20). The competitive index (CI) of the Δ*phcA* mutant was calculated as previously described (67).

Attachment of *R. solanacearum* to tomato roots was measured as previously described (82). Briefly, axenic tomato seedling roots were incubated 2 h in a dilute bacterial suspension, rinsed, blotted to remove unattached cells, and then ground and dilution plated to quantify attached bacteria. Percent attachment was defined as the ratio of CFU attached per centimeter of root to the total CFU in the inoculum. The experiment was repeated three times, with 8 to 10 technical replicates of 4 pooled roots each per treatment each time.

### Recombinant DNA techniques and mutagenesis.

To construct an *R. solanacearum* GMI1000 Δ*phcA* strain (*phcA*::Gm^r^), the regions flanking *phcA* were amplified with Phusion high-fidelity DNA polymerase (Finnzymes, Vantaa, Finland), and a gentamicin resistance cassette amplified from pUC18t miniTn7t-Gm (Novagen, Madison, WI) was inserted between the two fragments via splice by overlap extension (SOE) PCR. This construct replaced the wild-type locus. A complemented *phcA* mutant strain was constructed by inserting the complete *phcA* open reading frame plus 227 bp of 5′ predicted promoter sequence into the selectively neutral chromosomal *att* site of the Δ*phcA* mutant strain using pRCT-tet without the Gateway cassette (83). Total genomic DNA and plasmid DNA were isolated, and *E. coli* and *R. solanacearum* were transformed as previously described (38). Primer sequences are listed in Table S4.

### RNA extraction for RNA-seq and library preparation.

Twenty-one-day-old tomato plants were inoculated with approximately 2,000 cells of either wild-type *R. solanacearum* strain GMI1000 or the Δ*phcA* mutant through a cut petiole as described above. After 72 h, 0.1 g of stem tissue spanning the inoculated petiole was placed in a tube containing 900 µl of prechilled transcription stop solution (5% [vol/vol] water-saturated phenol–ethanol) and ground using a Powerlyzer (Mo Bio Laboratories, Carlsbad, CA) for 1.5 cycles of 90 s each. Between grinding cycles, the rotor and tubes were placed on ice to minimize sample heating. The homogenate was centrifuged for 7 min in prechilled 1.5-ml tubes at 20,817 × *g*, supernatant was discarded, and the pellet was frozen in liquid nitrogen and stored at −80°C. RNA was extracted using the modified hot-phenol method as previously described (29). The RNA concentration and purity were determined by the use of a Nanodrop instrument (Thermo Fisher Scientific, Wilmington, DE), and sample quality was assessed using an Agilent Bioanalyzer 2100 Nanochip instrument (Agilent Technologies, Santa Clara, CA). Each biological replicate contained RNA pooled from six infected plants. The RNA-seq experiment included three biological replicates per treatment.

Library preparation and sequencing were performed by Protein CT Biotechnologies (Madison, WI). Briefly, 850 ng total RNA was treated with RiboZero kits (Epicentre, Madison, WI) to reduce bacterial and plant rRNA levels, followed by tomato mRNA reduction using poly(A) selection, and was used to make libraries with a Illumina TruSeq strand-specific mRNA sample preparation system. After RNA fragmentation, strand-specific libraries were constructed by first-strand cDNA synthesis using random primers, sample cleanup, second-strand synthesis, and DNA polymerase I and RNase H. A single “A” base was added to the cDNA fragments followed by ligation of the adapters. The final cDNA library was further purified and enriched with PCR and was quality checked using a Bioanalyzer 2100 instrument. The libraries were sequenced using an Illumina HiSeq 2500 system, yielding ~20 million 1× 50-bp reads per sample.

### Data quality check and analysis.

Illumina read raw data quality was verified with fastQC. The following genomes were used for mapping: the ITAG2.4 tomato genome (*S_lycopersicum*_chromosomes. 2.50.fa) and the *R. solanacearum* strain GMI1000 genome (http://www.ncbi.nlm.nih.gov/nuccore/NC_003295.1 and http://www.ncbi.nlm.nih.gov/nuccore/NC_003296.1). Raw sequence reads were mapped to genomes using the Subjunc aligner from Subread; most reads (>90% for all samples) aligned to the combined genomes, with about 1.2 to 4.5% total (84) reads per sample aligned to the bacterial genome. Bacterial alignment bam files were compared to the gene annotation GFF file, and raw counts for each gene were generated using the Subread featureCounts tool. Raw count data were normalized using the R Limma voom package (85) and then analyzed for differential expression. Blast2GO (86) was used for further data analysis and presentation.

### Metabolomic analysis of xylem sap.

Tomato xylem sap was harvested by root pressure. At 1 to 3 h after light onset, well-watered 26- to 28-day-old plants were detopped with a sharp blade. The sap that accumulated in the first 3 min was discarded, the stump was rinsed with water, and then sap was collected for 30 min. Five sap samples, each containing sap pooled from 4 plants, were analyzed. Samples were shipped on dry ice to the West Coast Metabolomics Center (Davis, CA) for gas chromatography-mass spectrometry (GC-MS) analysis. Metabolites were detected on a Pegasus IV GC instrument (Leco Corp., St. Joseph, MI) coupled to a Pegasus IV time of flight-MS (TOF-MS) system as previously described (87).

### Nitrite measurement.

Nitrite was quantified both *in vitro* and *in planta* as previously described (38). The *in vitro* experiment included three biological replicates with three technical replicates each. For *in planta* measurements, homogenized stems of uninfected plants were used as controls. Nitrite concentrations were calculated on a per-cell basis. Fourteen plants were sampled for each treatment.

### Biofilm formation.

Biofilm assays and confocal microscopy of *R. solanacearum* biofilm were performed as previously described (46). Confocal images were analyzed using Comstat2 (88).

### Scanning electron microscopy to visualize *R. solanacearum* in tomato xylem.

Scanning electron microscopy was performed as previously described (46). The experiment was repeated twice with a minimum of three plants per treatment. Two sections from each plant were visualized using a LEO 1530 scanning electron microscope at the University of Wisconsin—Madison (UW-Madison) Material Sciences Center.

### qRT-PCR.

Expression of selected plant and bacterial genes was measured by quantitative reverse transcriptase PCR (qRT-PCR) as previously described (29). The threshold cycle (ΔΔ*C*_*T*_) method was used to calculate log fold expression changes; data reflect three biological replicates, with two technical replicates each. Primers used for qPCR are listed in Table S4.

### Accession number(s).

The complete experimental data set was deposited in the Gene Expression Omnibus (GEO) database under GenBank accession number GSE98074.
