# Supplementary material for: A Single Regulator Mediates Strategic Switching between Attachment/Spread and Growth/Virulence in the Plant Pathogen Ralstonia solanacearum
Source: mBio. 2017 Sep 26;8(5):e00895-17. doi: 10.1128/mBio.00895-17 (PMC5615195; doi:10.1128/mBio.00895-17)
Supplement: TABLE S1 [file mbo005173501st1.pdf]

Table S1

A: Complete list of *R. solanacearum* strain GMI1000 genes differentially expressed in planta with >2 fold-change and a false discovery rate (FDR) ≤ 0.005

Gene [a]	Fold change [b]	Adj.P. Value [c]	Locus_tag [d]	Annotation [e]
RSp1618	42.3	2.09E-04	RS_RS24875	transcriptional regulator
RSc1737	35.5	4.50E-07	RS_RS08775	porin
RSp1468	24.3	2.64E-06	RS_RS24195	hypothetical protein
RSc2937	22.5	1.71E-08	RS_RS14715	iron ABC transporter substrate-binding protein
RSc2938	20.9	3.15E-09	RS_RS14720	spermidine/putrescine ABC transporter permease
RSc1750	18.2	7.57E-08	RS_RS08840	*livJ*; branched-chain amino acid ABC transporter substrate-binding protein
RSp1459	17.8	2.64E-06	RS_RS24150	hypothetical protein
RSc2939	15.6	4.91E-08	RS_RS14725	lipase
RSc2796	14.1	3.55E-05	RS_RS13980	hemagglutinin
RSc1733	13.9	4.91E-08	RS_RS08755	spermidine/putrescine ABC transporter substrate-binding protein
RSp0287	11.5	8.20E-07	RS_RS18540	*hmsF*; poly-beta-1,6-N-acetyl-D-glucosamine N-deacetylase PgaB
RSp0286	10.1	6.62E-07	RS_RS18535	*hmsH*; poly-beta-1,6 N-acetyl-D-glucosamine export porin PgaA
RSc1832	10.0	8.50E-06	RS_RS09240	transcriptional regulator
RSp0243	10.0	5.47E-05	RS_RS18325	heme ABC transporter
RSc1082	9.8	9.69E-06	RS_RS05375	porin
RSc0800	9.8	1.40E-06	RS_RS04020	ferrisiderophore receptor signal peptide protein
RSp0285	9.5	1.05E-05	RS_RS18530	LuxR family transcriptional regulator
RSc2797	9.4	2.08E-03	RS_RS13985	hemagglutinin
RSp0055	9.3	2.89E-04	RS_RS17460	*glyA2*; serine hydroxymethyltransferase
RSp0056	8.8	6.50E-05	RS_RS17465	peptidase M19
RSc2798	8.8	8.58E-05	RS_RS13990	hypothetical protein
RSp0795	8.5	6.47E-07	RS_RS20915	alpha/beta hydrolase
RSc1734	8.5	5.24E-06	RS_RS08760	Fe3+/spermidine/putrescine ABC transporter ATP-binding protein
RSp0828	8.2	9.85E-07	RS_RS21080	*gudP*; glucarate transporter
RSp0054	8.0	3.48E-05	RS_RS17455	AraC family transcriptional regulator
RSp1062	7.9	7.77E-06	RS_RS22260	TonB-dependent receptor
RSp0829	7.9	1.14E-05	RS_RS21085	*gudD2*; glucarate dehydratase
RSp1595	7.9	5.36E-04	RS_RS24770	3-(3-hydroxy-phenyl)propionate transporter MhpT
RSp0826	7.6	1.21E-06	RS_RS21070	5-dehydro-4-deoxyglucarate dehydratase
RSc1799	7.4	5.39E-04	RS_RS09080	branched-chain alpha-keto acid dehydrogenase subunit E2
RSc0801	7.3	2.06E-06	RS_RS04025	membrane protein
RSp0227	7.2	1.21E-05	RS_RS18245	*fcs*; feruloyl-CoA synthase
RSp0064	7.2	2.93E-05	RS_RS17505	*opuC*; glycine/betaine ABC transporter substrate-binding protein
RSc1081	7.1	1.05E-05	RS_RS05370	alpha-glucosidase
RSc0029	7.0	1.06E-05	RS_RS00145	*goaG*; 4-aminobutyrate transaminase
RSc1083	7.0	1.76E-03	RS_RS05380	aldose epimerase

RSc3127	6.9	5.19E-05	RS_RS15710	*eutB*; ethanolamine ammonia lyase large subunit
RSc1736	6.7	2.91E-03	RS_RS08770	DNA-binding response regulator
RSc1079	6.7	3.36E-07	RS_RS05360	*gudD1*; glucarate dehydratase
RSp0244	6.7	2.21E-05	RS_RS18330	membrane protein
RSc1751	6.4	1.45E-04	RS_RS08845	*livH1*; ABC transporter permease
RSc0802	6.4	1.76E-06	RS_RS04030	membrane protein
RSc1749	6.4	1.32E-06	RS_RS08835	*alkK*; long-chain-fatty-acid--CoA ligase
RSp0288	6.4	2.77E-05	RS_RS18545	*hmsR*; poly-beta-1,6 N-acetyl-D-glucosamine synthase
RSp1238	6.4	8.50E-04	RS_RS23115	polyisoprenoid-binding protein
RSp0831	6.3	9.15E-06	RS_RS21095	NAD-dependent dehydratase
RSp1619	6.2	2.35E-04	RS_RS24880	phosphoadenosine phosphosulfate sulfotransferase
RSc1080	6.1	4.96E-06	RS_RS05365	*exuT2*; hexuronate transporter ExuT
RSp0274	6.1	4.45E-06	RS_RS18475	*ggt1*; gamma-glutamyltransferase
RSp0830	5.9	4.96E-06	RS_RS21090	*garD*; galactarate dehydratase
RSp1098	5.7	1.40E-06	RS_RS22420	endonuclease
RSc0547	5.7	1.51E-05	RS_RS02740	MFS transporter
RSp0228	5.6	2.07E-05	RS_RS18250	sigma-54-dependent Fis family transcriptional regulator
RSp0289	5.4	4.19E-05	RS_RS18550	hypothetical protein
RSp0827	5.3	3.91E-06	RS_RS21075	2,5-dioxovalerate dehydrogenase
RSc1732	5.3	1.07E-05	RS_RS08750	polyamine ABC transporter substrate-binding protein
RSc1202	5.2	6.08E-05	RS_RS06020	alkene reductase
RSc1754	5.2	3.76E-04	RS_RS08860	*livF*; ABC transporter ATP-binding protein
RSp0972	5.2	4.53E-04	RS_RS21805	*narK1*; MFS transporter
RSc2725	5.1	7.49E-04	RS_RS13635	MarR family transcriptional regulator
RSp0417	5.1	3.37E-05	RS_RS19170	*cysK2*; siderophore biosynthesis protein SbnA
RSc1752	5.1	1.35E-04	RS_RS08850	*livM*; ABC transporter permease
RSc1245	5.0	2.13E-05	RS_RS06240	*iolI*; protein implicated IN myo-inositol catabolite pathway
RSc1239	5.0	8.30E-05	RS_RS06210	*iolD*; 3D-(3,5/4)-trihydroxycyclohexane-1,2-dione acylhydrolase
RSp1620	4.9	7.30E-04	RS_RS24885	hemagglutinin
RSc1241	4.9	4.30E-05	RS_RS06220	*mocB*; rhizopine-binding protein
RSp0832	4.9	1.90E-04	RS_RS21100	gluconolactonase
RSc3051	4.9	4.86E-06	RS_RS15330	ABC transporter substrate-binding protein
RSc3342	4.8	6.67E-06	RS_RS16745	ABC transporter permease
RSc1798	4.8	2.01E-03	RS_RS09075	2-oxoisovalerate dehydrogenase subunit beta
RSc1295	4.8	2.64E-03	RS_RS06490	MarR family transcriptional regulator
RSp0314	4.8	5.39E-04	RS_RS18655	DNA-binding response regulator
RSc3341	4.8	3.17E-05	RS_RS16740	ABC transporter permease
RSp0973	4.8	5.27E-04	RS_RS21810	*narK2*; nitrate transporter
RSc2238	4.7	6.10E-05	RS_RS11235	membrane protein
RSc2020	4.6	4.10E-05	RS_RS10150	acyl-CoA dehydrogenase
RSp0413	4.6	3.50E-03	RS_RS19150	transcriptional regulator

RSp0324	4.6	1.81E-03	RS_RS18705	(2Fe-2S)-binding protein
RSc3343	4.6	3.82E-05	RS_RS16750	ABC transporter ATP-binding protein
RSp1118	4.6	1.49E-04	RS_RS22515	hypothetical protein
RSc3139	4.6	9.06E-06	RS_RS15770	MarR family transcriptional regulator
RSc1244	4.5	2.73E-05	RS_RS06235	*iolH*; protein iolH
RSc2542	4.5	2.21E-03	RS_RS12745	membrane protein
RSc2963	4.5	3.86E-04	RS_RS14850	hypothetical protein
RSp0081	4.4	2.37E-05	RS_RS17580	N-acetyltransferase GCN5
RSp0814	4.4	1.75E-05	RS_RS21010	*mqo*; malate:quinone oxidoreductase
RSc0627	4.3	8.87E-05	RS_RS03150	aldehyde oxidase
RSp0403	4.3	2.86E-04	RS_RS19095	hypothetical protein
RSp1117	4.3	8.64E-05	RS_RS22510	hypothetical protein
RSp1389	4.3	3.02E-03	RS_RS23825	MBL fold metallo-hydrolase
RSc1203	4.3	9.06E-06	RS_RS06025	NADPH:quinone oxidoreductase
RSc0593	4.3	1.38E-04	RS_RS02970	hypothetical protein
RSc1242	4.3	1.51E-04	RS_RS06225	D-ribose transporter ATP-binding protein
RSc3047	4.3	1.63E-04	RS_RS15310	ABC transporter ATP-binding protein
RSp1458	4.3	1.63E-04	RS_RS24145	transporter protein
RSc1246	4.2	5.36E-05	RS_RS06245	*iolG*; inositol 2-dehydrogenase
RSp0316	4.2	4.57E-04	RS_RS18665	2-nitropropane dioxygenase
RSc0628	4.2	4.63E-04	RS_RS03155	lipoprotein
RSp0226	4.2	3.33E-05	RS_RS18240	*vdh*; salicylaldehyde dehydrogenase
RSp0422	4.2	4.52E-04	RS_RS19195	siderophore biosynthesis protein
RSc1753	4.2	8.63E-04	RS_RS08855	*livG*; ABC transporter ATP-binding protein
RSc1972	4.2	5.46E-05	RS_RS09910	*phaP*; phasin
RSc1622	4.2	3.81E-03	RS_RS08175	hypothetical protein
RSc3344	4.1	3.86E-05	RS_RS16755	ABC transporter ATP-binding protein
RSc2044	4.1	1.63E-04	RS_RS10255	acyl-CoA dehydrogenase
RSp0976	4.1	4.14E-04	RS_RS21825	*narJ*; nitrate reductase molybdenum cofactor assembly chaperone
RSc1247	4.1	2.69E-05	RS_RS06250	myo-inositol catabolism protein
RSp1119	4.1	4.95E-04	RS_RS22520	transcriptional regulator
RSc1084	4.0	5.54E-04	RS_RS05385	membrane protein
RSc1812	4.0	2.82E-03	RS_RS09145	hypothetical protein
RSp0421	4.0	1.02E-04	RS_RS19190	siderophore biosynthesis protein
RSp1614	4.0	1.00E-03	RS_RS24855	membrane protein
RSc1469	4.0	1.28E-03	RS_RS07395	carbon monoxide dehydrogenase
RSc1262	3.9	1.77E-04	RS_RS06325	hypothetical protein
RSc1236	3.9	4.00E-04	RS_RS06195	membrane protein
RSp0321	3.9	8.89E-04	RS_RS18690	hypothetical protein
RSp0420	3.9	1.37E-03	RS_RS19185	membrane protein
RSc0159	3.9	5.46E-05	RS_RS00785	ornithine--oxo-acid transaminase

RSp0053	3.9	7.89E-04	RS_RS17450	*fdhA*; formaldehyde dehydrogenase, glutathione-independent
RSc2012	3.8	3.59E-05	RS_RS10110	*gstH*; glutathione S-transferase
RSc1762	3.8	4.33E-04	RS_RS08900	acyl-CoA dehydrogenase
RSp0796	3.8	8.29E-04	RS_RS20920	transcription regulator protein
RSp0418	3.8	2.98E-05	RS_RS19175	2,3-diaminopropionate biosynthesis protein SbnB
RSp0484	3.8	3.35E-05	RS_RS19485	hypothetical protein
RSc1337	3.8	8.01E-04	RS_RS06705	FMN reductase (NADPH)
RSp0082	3.8	1.28E-03	RS_RS17585	hypothetical protein
RSp0080	3.8	1.29E-04	RS_RS17575	HxlR family transcriptional regulator
RSc0626	3.8	1.13E-03	RS_RS03145	(2Fe-2S)-binding protein
RSc1811	3.7	4.33E-04	RS_RS09140	short-chain isoprenyl diphosphate synthase
RSc1243	3.7	1.45E-04	RS_RS06230	sugar ABC transporter permease
RSc1380	3.7	1.36E-04	RS_RS06920	*gsiB*; glutathione ABC transporter substrate-binding protein
RSc1759	3.7	9.70E-06	RS_RS08885	3-hydroxyacyl-CoA dehydrogenase
RSp1615	3.7	2.87E-03	RS_RS24860	*uxaA*; galactarate dehydratase
RSp0225	3.7	5.39E-04	RS_RS18235	*fca*; crotonase
RSc0028	3.7	1.54E-04	RS_RS00140	*gabD1*; NAD-dependent succinate-semialdehyde dehydrogenase
RSp1396	3.7	1.15E-03	RS_RS23860	membrane protein
RSc1264	3.7	2.93E-05	RS_RS06335	*ugpB*; glycerol-3-phosphate ABC transporter substrate-binding protein
RSc1248	3.6	3.94E-05	RS_RS06255	oxidoreductase
RSp0974	3.6	2.51E-03	RS_RS21815	*narG*; nitrate reductase subunit alpha
rRNA-23S	3.6	3.50E-03	RS_RS15270	23S ribosomal RNA
RSp0424	3.6	1.75E-04	RS_RS19205	diaminopimelate decarboxylase
rRNA-23S	3.6	2.43E-03	RS_RS14365	23S ribosomal RNA
RSc1240	3.6	2.89E-04	RS_RS06215	*iolC*; 5-dehydro-2-deoxygluconokinase
RSc1340	3.6	2.13E-03	RS_RS06720	*ssuC*; sulfonate ABC transporter
RSp1592	3.6	5.71E-05	RS_RS24755	*livK*; branched chain amino acid ABC transporter substrate-binding
RSc1339	3.5	1.12E-03	RS_RS06715	*ssuD*; alkanesulfonate monooxygenase
RSc0624	3.5	1.86E-03	RS_RS03135	IMP dehydrogenase
RSc1336	3.5	9.36E-05	RS_RS06700	*sbp*; ABC transporter permease
RSc2043	3.5	7.80E-04	RS_RS10250	MaoC family dehydratase
RSc2045	3.4	6.08E-05	RS_RS10260	acyl-CoA dehydrogenase
RSc3059	3.4	8.50E-06	RS_RS15370	MFS transporter
RSc0297	3.4	1.62E-03	RS_RS01460	hypothetical protein
RSp0414	3.4	2.43E-04	RS_RS19155	ferric siderophore receptor protein
RSp0481	3.4	2.79E-04	RS_RS19470	ABC transporter substrate-binding protein
RSc3128	3.4	6.64E-05	RS_RS15715	*exaC*; aldehyde dehydrogenase
RSp1122	3.4	6.08E-05	RS_RS22535	hydrolase
RSc1267	3.4	1.65E-03	RS_RS06350	*upgC*; sn-glycerol-3-phosphate import ATP-binding protein UgpC
rRNA-16S	3.3	2.21E-03	RS_RS14380	16S ribosomal RNA
RSp0315	3.3	1.63E-04	RS_RS18660	two-component sensor histidine kinase

RSc1761	3.3	3.12E-04	RS_RS08895	acetyl-CoA acetyltransferase
RSp0975	3.3	4.47E-03	RS_RS21820	*narH*; nitrate reductase subunit beta
RSc1381	3.3	2.64E-03	RS_RS06925	*gsiC*; glutathione ABC transporter permease GsiC
RSc2253	3.3	1.02E-04	RS_RS11310	*pcaJ*; 3-oxoadipate CoA-transferase subunit B
RSc1266	3.3	2.21E-03	RS_RS06345	*ugpE*; glycerol-3-phosphate transporter membrane protein
RSp0977	3.3	6.62E-04	RS_RS21830	*narI*; nitrate reductase
RSp0996	3.2	2.25E-03	RS_RS21930	*ttuC2*; tartrate dehydrogenase
RSc2376	3.2	4.04E-03	RS_RS11930	4Fe-4S ferredoxin
RSc1697	3.2	3.23E-04	RS_RS08570	DNA repair protein HhH-GPD
RSc0780	3.2	5.53E-04	RS_RS03920	*fadD2*; long-chain-fatty-acid--CoA ligase
RSp0051	3.2	2.12E-03	RS_RS17440	porin
RSp1120	3.2	3.64E-03	RS_RS22525	hypothetical protein
RSc0473	3.2	1.87E-04	RS_RS02345	acyl-CoA dehydrogenase
RSc0484	3.2	3.61E-05	RS_RS02400	*gltL*; arginine ABC transporter ATP-binding protein
RSc1763	3.2	3.64E-04	RS_RS08905	gluconate 2-dehydrogenase
RSc2254	3.2	1.15E-03	RS_RS11315	*pcaI*; 3-oxoadipate CoA-transferase subunit A
RSp1159	3.1	3.77E-04	RS_RS22720	universal stress protein UspA
RSc1265	3.1	6.72E-04	RS_RS06340	*ugpA*; glycerol-3-phosphate transporter permease
RSc0481	3.1	2.41E-04	RS_RS02385	ABC transporter
RSc2799	3.1	3.51E-03	RS_RS13995	membrane protein
RSp0419	3.1	2.66E-03	RS_RS19180	siderophore biosynthesis protein
RSc1238	3.1	1.43E-04	RS_RS06205	*iolE*; myo-inosose-2 dehydratase
RSc3048	3.1	2.13E-03	RS_RS15315	ABC transporter permease
RSc1463	3.1	1.28E-03	RS_RS07365	hypothetical protein
RSp0107	3.1	2.29E-04	RS_RS17665	MFS transporter
RSc1731	3.1	4.80E-04	RS_RS08745	polyamine ABC transporter permease
RSp1591	3.0	1.28E-03	RS_RS24750	aldehyde dehydrogenase
RSc3270	3.0	4.73E-03	RS_RS16380	hypothetical protein
RSc1442	3.0	7.05E-04	RS_RS07260	*pcaG*; protocatechuate 3,4-dioxygenase subunit alpha
RSc3075	3.0	3.65E-03	NA	NA
RSp1047	3.0	2.12E-03	RS_RS22185	MFS transporter
RSp0048	2.9	7.30E-04	RS_RS17425	*soxA1*; sarcosine oxidase subunit alpha
RSc1201	2.9	7.55E-05	RS_RS06015	TetR family transcriptional regulator
RSc1769	2.9	4.68E-03	RS_RS08935	hypothetical protein
RSc2431	2.9	4.59E-04	RS_RS12205	chloride channel protein
RSc0476	2.9	5.22E-04	RS_RS02360	enoyl-CoA hydratase
RSc2095	2.9	3.64E-03	RS_RS10510	*xdhA*; xanthine dehydrogenase small subunit
RSp0273	2.9	1.49E-03	RS_RS18465	glutamate carboxypeptidase
RSc2042	2.9	9.40E-04	RS_RS10245	MaoC family dehydratase
RSc0746	2.8	2.44E-03	RS_RS03760	*tauD*; taurine dioxygenase
RSc1562	2.8	2.83E-03	RS_RS07865	O-acetylhomoserine aminocarboxypropyltransferase

RSc2046	2.8	4.48E-04	RS_RS10265	hypothetical protein
RSp1623	2.8	1.44E-03	RS_RS24900	histidine kinase
RSp0320	2.8	1.37E-03	RS_RS18685	Cd(II)/Pb(II)-responsive transcriptional regulator
RSc1760	2.8	1.13E-03	RS_RS08890	*pncA*; bifunctional pyrazinamidase/nicotinamidase hydrolase
RSc2647	2.7	8.94E-04	RS_RS13255	*hutU*; urocanate hydratase
RSc3045	2.7	7.92E-04	RS_RS15300	*glpD*; glycerol-3-phosphate dehydrogenase
RSc1468	2.7	2.15E-03	RS_RS07390	carbon monoxide dehydrogenase
RSc0474	2.7	1.38E-04	RS_RS02350	3-hydroxyacyl-CoA dehydrogenase
RSc0483	2.7	9.33E-05	RS_RS02395	*gltK*; glutamate/aspartate transporter permease GltK
RSp0850	2.7	3.10E-03	RS_RS21195	*prhR*; iron dicitrate transporter FecR
RSc3329	2.7	3.88E-04	RS_RS16680	branched-chain amino acid ABC transporter substrate-binding protein
RSc2151	2.6	1.78E-03	RS_RS10785	hypothetical protein
RSp0849	2.6	9.70E-04	RS_RS21190	*prhI*; RNA polymerase sigma factor
RSp0530	2.6	4.33E-04	RS_RS19710	cytochrome-c peroxidase
RSc2257	2.6	7.59E-05	RS_RS11330	amino acid ABC transporter substrate-binding protein
RSc0475	2.6	3.63E-04	RS_RS02355	acetyl-CoA acetyltransferase
RSp0483	2.6	2.13E-03	RS_RS19480	ornithine cyclodeaminase
RSc0482	2.5	3.04E-04	RS_RS02390	*gltJ*; glutamate/aspartate ABC transporter permease GltJ
RSc1975	2.5	1.00E-03	RS_RS09925	multidrug ABC transporter permease
RSc2353	2.5	2.68E-03	RS_RS11815	hypothetical protein
RSp1113	2.5	9.14E-04	RS_RS22490	multidrug efflux RND transporter permease subunit
RSc2021	2.5	2.93E-04	RS_RS10155	thiol:disulfide oxidoreductase
RSp1670	2.5	4.06E-03	RS_RS25115	hypothetical protein
RSc2014	2.5	4.69E-03	RS_RS10120	*paaG2*; enoyl-CoA hydratase
RSp1590	2.5	1.24E-03	RS_RS24745	FAD-dependent oxidoreductase
RSp0242	2.5	3.81E-03	RS_RS18320	*glgA*; glycogen synthase
RSc3392	2.5	3.45E-03	RS_RS16995	DNA-binding response regulator
RSc0981	2.5	5.64E-04	RS_RS04855	*lldP*; lactate permease
RSc1293	2.4	3.86E-03	RS_RS06480	*emrA*; hemolysin D
RSc1567	2.4	5.18E-04	RS_RS07890	dehydrogenase
RSp0079	2.4	3.73E-03	RS_RS17570	GntR family transcriptional regulator
RSc1342	2.3	3.04E-03	RS_RS06730	*ssuF*; transporter
RSp0993	2.3	4.00E-03	RS_RS21915	membrane protein
RSc3386	2.3	3.01E-03	NA	NA
RSc1316	2.2	1.89E-03	RS_RS06600	phosphate starvation protein PhoH
RSc1014	2.2	7.63E-04	RS_RS05020	LacI family transcriptional regulator
RSc2512	2.2	1.93E-03	RS_RS12595	arogenate dehydratase
RSc0010	2.2	5.88E-04	RS_RS00045	*acrB*; multidrug efflux RND transporter permease subunit
RSp0676	2.2	1.94E-03	RS_RS20360	metE 5-methyltetrahydropteroyltriglutamate-- homocysteine methyltransferase
RSc2244	2.2	4.02E-03	RS_RS11265	ABC transporter substrate-binding protein
RSc1008	2.2	4.43E-04	RS_RS04990	*phaZ*; esterase

RSp1048	2.2	8.49E-04	RS_RS22190	formate dehydrogenase oxidoreduct
RSc2041	2.2	3.15E-03	RS_RS10240	urea ABC transporter substrate-binding protein
RSc2422	2.1	3.53E-03	RS_RS12155	*cysD*; sulfate adenylyltransferase small subunit
RSp1308	2.1	3.74E-03	RS_RS23460	carboxymethylenebutenolidase
RSc2425	2.1	3.30E-03	RS_RS12170	*cysI1*; sulfite reductase
RSc0074	2.1	4.00E-03	RS_RS00370	*htpX*; protease HtpX homolog
RSc1766	2.1	3.21E-03	RS_RS08920	NADP-dependent oxidoreductase
RSc1563	2.1	2.84E-03	RS_RS07870	CBS domain-containing protein
RSc1231	2.1	6.47E-04	RS_RS06170	*tal*; transaldolase
RSc0472	2.1	4.47E-03	RS_RS02340	TetR family transcriptional regulator
RSp0013	2.0	3.19E-03	RS_RS17260	amino acid ABC transporter substrate-binding protein
RSc0009	2.0	4.11E-03	RS_RS00040	multidrug transporter
RSp0416	2.0	2.83E-03	RS_RS19165	membrane protein
RSc2421	2.0	3.60E-03	RS_RS12150	*cysN*; sulfate adenylyltransferase

Downregulated genes

**Gene** [a]	**Fold change** [b]	**Adj.P. Value** [c]	**Locus_tag** [d]	**Annotation** [e]
RSc1727	-261.9	1.55E-06	RS_RS08725	hypothetical protein
RSp0583	-228.6	2.42E-09	NA	NA
RSp1021	-226.3	2.64E-06	NA	NA
RSc1728	-224.3	2.80E-05	RS_RS08730	radical SAM/SPASM maturase
RSp1016	-203.1	2.48E-07	RS_RS22025	*wecC*; NDP-N-acetyl-D-galactosaminuronic acid dehydrogenase
RSp1002	-200.3	1.51E-09	RS_RS21955	*tek*; TEK signal peptide protein
RSc2107	-192.3	2.62E-08	RS_RS10570	hypothetical protein
RSp0169	-171.1	3.15E-09	RS_RS17960	membrane protein
RSc1723	-160.5	4.96E-06	NA	NA
RSp0569	-129.1	2.55E-07	NA	NA
RSp1014	-122.8	4.96E-06	RS_RS22015	*epsF*; EPS I polysaccharide export inner membrane protein EpsF
RSp1020	-113.0	3.36E-07	RS_RS22045	*epsA*; EPS I polysaccharide export outer membrane protein EpsA
RSp1015	-112.3	4.26E-06	RS_RS22020	*epsE*; EPS I polysaccharide export inner membrane protein EpsE
RSp0982	-108.9	1.91E-05	RS_RS21855	hypothetical protein
RSp1018	-101.7	8.62E-08	RS_RS22035	*epsB*; tyrosine protein kinase
RSp1003	-99.6	7.57E-08	RS_RS21960	*xpsR*; transcriptional regulator
RSp1019	-98.9	2.80E-05	RS_RS22040	*epsP*; protein-tyrosine-phosphatase
RSc1725	-92.0	6.47E-07	RS_RS08715	hypothetical protein
RSp1010	-90.9	1.21E-06	RS_RS21995	hypothetical protein
RSp0162	-90.5	4.00E-07	RS_RS17915	*egl*; drug:proton antiporter
RSp1017	-89.3	1.34E-06	RS_RS22030	*epsC*; UDP-N-acetyl glucosamine 2-epimerase
RSc0232	-86.6	2.92E-06	RS_RS01140	hypothetical protein
RSp1091	-81.1	9.64E-06	RS_RS22385	*cpaA2*; peptidase
RSp1090	-78.8	1.85E-05	RS_RS22380	pilus biosynthesis protein TadE

RSp1009	-74.3	1.65E-06	RS_RS21990	glycosyltransferase WbuB
RSc2026	-73.1	1.13E-05	NA	NA
RSp1004	-71.0	5.04E-07	RS_RS21965	dTDP-glucose 4,6-dehydratase
RSc3288	-69.7	2.48E-07	RS_RS16475	sugar-binding lectin protein
RSc0025	-69.5	1.16E-07	RS_RS00125	hypothetical protein
RSp0174	-67.4	2.14E-06	RS_RS17980	methyltransferase
RSp1417	-66.9	1.33E-06	RS_RS23960	MFS transporter
RSc1726	-63.6	2.48E-07	RS_RS08720	hypothetical protein
RSp1013	-60.6	6.83E-07	RS_RS22010	transferase
RSc1910	-58.5	5.65E-05	NA	NA
RSc2508	-57.0	2.64E-08	RS_RS12575	toxin HipA
RSp0173	-54.5	5.54E-06	RS_RS17975	NIFS-like protein
RSp1434	-50.3	1.09E-05	RS_RS24040	amino acid adenylation protein
RSp1011	-47.9	4.59E-07	RS_RS22000	dehydrogenase
RSp1493	-46.6	6.96E-05	NA	NA
RSp1005	-42.0	1.80E-06	RS_RS21970	membrane protein
RSc3292	-41.4	7.15E-05	RS_RS16495	membrane protein
RSp0999	-40.2	1.04E-04	NA	NA
RSp1087	-39.7	2.21E-05	RS_RS22365	membrane protein
RSp0602	-37.9	2.64E-08	RS_RS19995	hypothetical protein
RSp0603	-37.7	4.91E-08	RS_RS20000	serine protease
RSp1092	-37.7	2.21E-05	RS_RS22390	pilus assembly protein
RSp1492	-36.4	9.30E-06	RS_RS24315	hypothetical protein
RSp1422	-36.1	6.03E-05	RS_RS23980	non-ribosomal peptide synthetase
RSc2025	-35.9	6.44E-07	RS_RS10175	hypothetical protein
RSp1603	-35.5	3.08E-04	RS_RS24800	calcium-binding ef-hand hnh nuclease
RSc2027	-35.1	1.72E-05	NA	NA
RSp1437	-33.4	1.45E-04	RS_RS25215	hypothetical protein
RSp1654	-33.2	1.95E-06	RS_RS25045	hypothetical protein
RSp0743	-33.2	1.16E-05	RS_RS20670	type VI secretion protein
RSp1430	-32.9	3.94E-05	RS_RS24020	hypothetical protein
RSp0501	-32.6	3.04E-05	RS_RS19570	hypothetical protein
RSp1263	-32.4	3.62E-07	RS_RS23235	*nadB2*; L-aspartate oxidase
RSc0582	-32.2	2.80E-05	RS_RS02915	avrd-related protein
RSp1419	-31.7	1.45E-07	RS_RS23970	peptide synthetase
RSp1527	-31.5	1.26E-04	RS_RS24485	membrane protein
RSp1436	-31.3	3.51E-05	RS_RS24050	acetyltransferase
RSp1418	-31.1	1.82E-06	RS_RS23965	hydrolase
RSp1486	-30.8	1.34E-06	RS_RS24290	hypothetical protein
RSp1012	-29.7	2.80E-05	RS_RS22005	acyltransferase
RSp0172	-29.2	5.24E-06	RS_RS17970	hypothetical protein

RSp1023	-29.1	7.55E-06	RS_RS22060	hypothetical protein
RSp0532	-28.6	5.54E-06	RS_RS19720	peptidase M1
RSp1656	-28.5	2.66E-04	RS_RS25055	ferritin
RSp0779	-28.1	6.30E-05	RS_RS20835	N-acylhomoserine lactone synthase
RSp0957	-26.6	1.75E-05	RS_RS21730	hypothetical protein
RSc2024	-26.3	6.08E-05	RS_RS10170	amino acid transporter
RSp1075	-25.1	1.55E-05	RS_RS22305	membrane protein
RSp1138	-25.1	3.09E-04	RS_RS22610	hypothetical protein
RSp0745	-25.0	3.36E-07	RS_RS20680	hypothetical protein
RSp0924	-24.2	4.35E-06	RS_RS21540	hypothetical protein
RSc2758	-23.9	1.07E-04	RS_RS13800	*araF*; arabinose ABC transporter substrate-binding protein
RSp1085	-23.7	7.05E-05	RS_RS22355	*cpaF2*; pilus assembly protein CpaF
RSp1000	-23.5	2.49E-07	RS_RS21945	*tek2*; TEK signal peptide protein
RSp0640	-23.3	1.99E-04	NA	NA
RSp0198	-23.1	4.19E-05	NA	NA
RSp1602	-22.9	3.13E-04	NA	NA
RSp0781	-22.4	1.13E-05	RS_RS20845	*metB*; cystathionine gamma-synthase
RSp0767	-22.3	3.18E-04	RS_RS20790	membrane protein
RSp0747	-21.9	1.07E-05	RS_RS20690	type VI secretion protein
RSp1487	-21.8	1.45E-07	RS_RS24295	*cmcH*; proline dehydrogenase
RSp1438	-21.7	4.33E-04	RS_RS24060	methionine--tRNA ligase
RSp1001	-21.6	2.46E-06	RS_RS21950	hypothetical protein
RSc3287	-21.4	9.00E-06	RS_RS16470	*solR*; transcriptional activator protein SolR
RSp1526	-20.8	3.32E-05	RS_RS24480	hypothetical protein
RSc0094	-20.7	8.50E-04	RS_RS00475	membrane protein
RSp1529	-20.6	2.37E-05	RS_RS24495	2-oxoglutarate-dependent ethylene/succinate-forming enzyme
RSp1424	-20.5	1.02E-04	RS_RS23990	*ectB*; diaminobutyrate--2-oxoglutarate transaminase
RSc1817	-20.2	4.65E-06	RS_RS09165	siderophore biosynthesis protein
RSp1429	-20.1	1.29E-04	RS_RS24015	hypothetical protein
RSp0742	-20.1	4.90E-06	RS_RS20665	hypothetical protein
RSp0746	-19.9	6.08E-05	RS_RS20685	type VI secretion protein
RSp0170	-19.7	1.01E-05	NA	NA
RSp0727	-19.6	1.28E-03	RS_RS20600	XRE family transcriptional regulator
RSp1086	-19.5	5.28E-04	RS_RS22360	*cpaE2*; pilus assembly protein
RSp1428	-19.5	1.60E-05	RS_RS24010	rhodanese
RSp1440	-19.5	4.24E-04	NA	NA
RSc1908	-19.5	2.21E-05	RS_RS09625	hypothetical protein
RSc2509	-19.4	2.01E-06	RS_RS12580	hypothetical protein
RSp1416	-19.3	3.92E-05	RS_RS23955	membrane protein
RSp1425	-19.3	3.26E-05	RS_RS23995	*pvdA*; ornithine monooxygenase
RSp0741	-19.1	1.14E-03	RS_RS20660	membrane protein

RSp0261	-18.9	8.50E-06	RS_RS18415	membrane protein
RSp0981	-18.8	2.81E-04	RS_RS21850	hypothetical protein
RSp0500	-18.2	2.49E-05	RS_RS19565	hypothetical protein
RSp1006	-18.2	1.70E-06	RS_RS21975	aminotransferase
RSp0766	-18.0	2.60E-04	RS_RS20785	hypothetical protein
RSp0199	-17.8	3.63E-06	RS_RS18100	disulfide bond formation protein B
RSp1089	-17.4	3.21E-04	RS_RS22375	pilus assembly protein CpaB
RSp0304	-17.3	1.28E-06	RS_RS18605	hypothetical protein
RSp1007	-17.3	2.02E-05	RS_RS21980	acetyltransferase
RSp1025	-17.2	1.86E-06	RS_RS22075	membrane protein
RSp1489	-17.2	2.21E-05	RS_RS24300	hypothetical protein
RSp1604	-17.2	1.18E-03	RS_RS24805	membrane protein
RSp1427	-17.0	1.06E-05	RS_RS24005	hypothetical protein
RSc0095	-17.0	3.22E-03	NA	NA
RSc0118	-17.0	6.13E-04	RS_RS00580	hypothetical protein
RSp1488	-16.5	2.87E-04	NA	NA
RSp1181	-16.5	4.57E-04	RS_RS22830	transporter
RSp1008	-16.1	1.92E-04	RS_RS21985	sugar transferase
RSp0163	-16.1	1.28E-04	RS_RS17920	4'-phosphopantetheinyl transferase
RSc1909	-16.0	1.72E-04	NA	NA
RSp1572	-15.9	5.08E-05	RS_RS24680	MFS transporter
RSp0535	-15.8	7.35E-04	RS_RS19735	LysR family transcriptional regulator
RSp0200	-15.6	6.38E-05	RS_RS18105	aminotransferase
RSc2108	-15.6	2.98E-05	RS_RS10575	porin
RSp0568	-15.5	3.38E-05	RS_RS19860	hypothetical protein
RSp0956	-15.4	2.29E-04	RS_RS21725	hypothetical protein
RSc2286	-15.3	1.75E-04	RS_RS11470	hypothetical protein
RSp1491	-15.2	1.95E-05	RS_RS24310	hypothetical protein
RSc0613	-15.0	2.23E-04	RS_RS03075	hypothetical protein
RSc2751	-15.0	5.28E-04	RS_RS13765	*dgoAb*; D-galactonate dehydratase
RSp0726	-14.9	7.62E-05	RS_RS20595	MFS transporter
RSc1494	-14.7	7.20E-04	RS_RS07510	integrase
RSp1652	-14.7	6.56E-04	RS_RS25035	serine/threonine protein phosphatase
RSc1816	-14.2	2.45E-04	RS_RS09160	acetyltransferase
RSp0749	-13.9	1.13E-05	RS_RS20700	ClpV1 family T6SS ATPase
RSc2759	-13.8	6.05E-04	RS_RS13805	*dgoK*; 2-dehydro-3-deoxygalactonokinase
RSp0338	-13.7	7.05E-05	RS_RS18775	*epsR*; multidrug MFS transporter
tRNA-serW	-13.6	1.59E-03	RS_RS09575	tRNA-Ser
RSc2757	-13.6	1.01E-03	RS_RS13795	*araG*; arabinose import ATP-binding protein AraG
RSp0519	-13.5	2.64E-03	RS_RS19655	signal transduction ggdef domain protein
RSc1729	-13.4	3.91E-03	RS_RS08735	LysR family transcriptional regulator

RSp0725	-13.3	3.38E-05	RS_RS20590	radical SAM protein
RSc2285	-13.3	1.72E-05	RS_RS11465	signal peptidase
RSp1439	-13.0	2.95E-05	RS_RS24065	cysE1; serine acetyltransferase
RSp1024	-12.7	4.50E-07	RS_RS22070	AWR family protein
RSp0986	-12.6	1.49E-03	RS_RS21875	*gbd2*; 4-hydroxybutyrate dehydrogenase
RSp1610	-12.5	2.11E-06	RS_RS24835	hypothetical protein
RSp0785	-12.4	1.28E-03	RS_RS20865	hypothetical protein
RSp1655	-12.3	2.67E-03	RS_RS25050	hypothetical protein
RSc3146	-12.1	2.80E-05	RS_RS15805	hypothetical protein
RSp1605	-12.0	8.88E-06	RS_RS24810	hemagglutinin
RSp1571	-12.0	4.99E-05	RS_RS24675	transcriptional regulator
RSc1819	-11.9	2.87E-04	RS_RS09175	hypothetical protein
RSc1926	-11.9	1.69E-03	RS_RS09705	hypothetical protein
RSc0233	-11.8	2.50E-04	RS_RS01145	multidrug DMT transporter permease
RSp1137	-11.8	3.17E-05	RS_RS22605	hypothetical protein
RSp0738	-11.7	1.29E-04	RS_RS20645	type VI secretion protein
RSc3123	-11.7	5.94E-08	RS_RS15685	transposase
RSc1818	-11.6	1.28E-05	RS_RS09170	*metG2*; methionine--tRNA ligase
RSp0584	-11.5	4.33E-04	RS_RS19925	transposase
RSp0780	-11.5	3.40E-05	RS_RS20840	hypothetical protein
RSp0710	-11.4	6.34E-04	RS_RS20530	membrane protein
RSc0581	-11.3	3.59E-05	RS_RS02910	phosphoglycolate phosphatase, bacterial
RSp1485	-11.1	9.06E-06	RS_RS24285	hypothetical protein
RSp1528	-11.0	1.41E-03	RS_RS24490	hypothetical protein
RSc1546	-11.0	5.24E-06	RS_RS07775	formate acetyltransferase
RSp0275	-10.9	2.19E-05	RS_RS18480	membrane protein
RSp0883	-10.9	1.38E-03	RS_RS21360	hypothetical protein
RSp0748	-10.8	2.79E-04	RS_RS20695	type VI secretion protein
RSp0641	-10.7	1.68E-07	RS_RS20185	hybrid non-ribosomal peptide synthase/polyketide synthase
RSp0841	-10.7	1.05E-03	RS_RS21145	hypothetical protein
RSc3151	-10.7	3.64E-05	RS_RS15830	tryptophan 2-monooxygenase oxidoreductase
RSc2106	-10.7	4.04E-04	RS_RS10565	amino acid ABC transporter ATP-binding protein
RSc0590	-10.5	5.46E-05	RS_RS02955	MarR family transcriptional regulator
RSp0788	-10.5	6.82E-05	RS_RS20880	carbamoyltransferase
RSp0497	-10.5	6.34E-04	RS_RS19550	hypothetical protein
RSp0744	-10.4	4.00E-07	RS_RS20675	type VI secretion protein EvpB
RSp0498	-10.4	4.48E-04	RS_RS19555	methionine biosynthesis protein MetW
RSp1426	-10.3	1.74E-03	RS_RS24000	hypothetical protein
RSp0765	-10.2	1.54E-03	RS_RS20780	hypothetical protein
RSc2761	-10.2	1.52E-03	RS_RS13815	LysR family transcriptional regulator
RSp1432	-10.2	2.52E-04	RS_RS24030	hypothetical protein

RSp1530	-10.1	9.12E-04	RS_RS24500	L-ascorbate oxidase
RSp0768	-10.1	3.10E-04	RS_RS20795	type VI secretion protein
RSp1653	-9.8	3.86E-03	RS_RS25040	cysteine ABC transporter ATP-binding protein
RSc0616	-9.6	2.79E-04	RS_RS03090	hypothetical protein
RSp0777	-9.6	1.89E-03	NA	NA
RSp0787	-9.5	5.56E-04	RS_RS20875	membrane protein
RSp0567	-9.4	2.30E-03	RS_RS19855	transposase
RSp0499	-9.4	1.99E-04	RS_RS19560	MFS transporter
RSp1580	-9.3	2.21E-03	RS_RS24720	hypothetical protein
RSc0120	-9.3	4.45E-03	RS_RS00590	GNAT family N-acetyltransferase
RSp1058	-9.2	1.10E-03	RS_RS22240	*adc*; acetoacetate decarboxylase
RSp0736	-9.2	4.68E-04	NA	NA
RSp0496	-9.1	3.38E-05	RS_RS19545	hypothetical protein
RSp1059	-9.1	2.45E-04	RS_RS22245	3-hydroxybutyrate dehydrogenase
RSp0782	-9.0	9.08E-05	RS_RS20850	aminoacyl-tRNA synthet
RSc2181	-9.0	4.32E-04	RS_RS10945	radical SAM protein
RSp0638	-9.0	2.52E-04	RS_RS20175	ABC transporter ATP-binding protein
RSp1431	-8.9	4.69E-04	RS_RS24025	oxidoreductase signal peptide protein
RSp0639	-8.8	2.17E-04	RS_RS20180	polyketide synthase
RSc0116	-8.8	1.90E-03	RS_RS00570	hypothetical protein
RSp1483	-8.7	3.87E-04	RS_RS24275	hypothetical protein
RSc1547	-8.7	1.64E-06	RS_RS07780	hypothetical protein
RSc3153	-8.6	3.02E-03	RS_RS15840	hypothetical protein
RSp0790	-8.6	2.64E-03	RS_RS20890	hydrolase
RSp0693	-8.5	1.69E-03	RS_RS20445	lactoylglutathione lyase
RSp1139	-8.5	1.27E-05	RS_RS22615	type VI secretion protein
RSp0694	-8.3	3.80E-04	RS_RS20450	Tryptophan 2,3-dioxygenase 2
RSc3145	-8.3	3.38E-05	RS_RS15800	transposase
RSc0971	-8.2	9.69E-06	RS_RS04805	hypothetical protein
RSp1168	-8.2	3.29E-05	RS_RS22765	hypothetical protein
RSc2023	-8.1	4.04E-03	RS_RS10165	hypothetical protein
RSp1167	-8.1	3.47E-06	RS_RS22760	choline dehydrogenase
RSc1003	-8.1	4.01E-05	RS_RS04965	peptidase S1
RSp0750	-8.1	1.39E-04	RS_RS20705	type VI secretion protein
RSp0998	-7.9	2.44E-03	RS_RS21940	rhomboid family intramembrane serine protease
RSp1607	-7.6	1.12E-05	RS_RS24820	hypothetical protein
RSp0277	-7.5	1.03E-05	RS_RS18490	*treA*; periplasmic trehalase
RSp1490	-7.5	3.90E-03	RS_RS24305	hypothetical protein
RSp0212	-7.5	3.99E-04	RS_RS18170	carotenoid oxygenase
RSp0171	-7.5	1.00E-03	RS_RS17965	hypothetical protein
RSp1606	-7.2	4.04E-04	RS_RS24815	hypothetical protein

RSp0739	-7.2	4.68E-04	RS_RS20650	type VI secretion system protein ImpK
RSp0882	-7.1	1.09E-03	RS_RS21355	hypothetical protein
RSp0740	-7.1	8.83E-04	RS_RS20655	type VI secretion protein
RSp0786	-7.1	1.10E-03	RS_RS20870	beta-ketoacyl-ACP synthase
RSp1180	-7.1	2.47E-06	RS_RS22825	type I secretion C-terminal target domain-containing protein
RSp0495	-7.1	1.52E-03	RS_RS19540	hypothetical protein
RSc1944	-7.0	6.64E-04	RS_RS09785	type VI secretion protein
RSp0983	-7.0	1.02E-04	RS_RS21860	helix-turn-helix transcriptional regulator
RSc1856	-7.0	4.31E-03	RS_RS09370	signal peptidase
RSp1500	-6.8	2.94E-03	RS_RS24345	spore coat protein U
RSp1423	-6.8	4.33E-04	RS_RS23985	hypothetical protein
RSp0771	-6.8	7.20E-04	RS_RS20810	hypothetical protein
RSc1820	-6.6	1.46E-03	RS_RS09180	Asp/Glu/hydantoin racemase
RSp1433	-6.6	3.63E-03	RS_RS24035	hypothetical protein
RSp1484	-6.6	5.17E-05	RS_RS24280	hypothetical protein
RSc3101	-6.6	3.64E-05	RS_RS15575	serine protease
RSc2388	-6.5	2.87E-04	NA	NA
RSp1609	-6.5	1.03E-05	RS_RS24830	membrane protein
RSc1605	-6.4	4.32E-08	RS_RS08080	hypothetical protein
RSp1573	-6.4	6.34E-04	RS_RS24685	hypothetical protein
RSc0818	-6.4	1.05E-03	RS_RS04105	endoglucanase
RSp0278	-6.3	1.74E-04	RS_RS18495	hypothetical protein
RSp0737	-6.3	6.88E-04	NA	NA
RSp0310	-6.2	5.71E-05	RS_RS18635	membrane protein
RSp0840	-6.2	1.02E-03	RS_RS21140	membrane protein
RSc0617	-6.2	8.46E-04	RS_RS03095	signal peptidase
RSp0789	-6.1	3.06E-03	RS_RS20885	hypothetical protein
RSc1501	-6.1	3.56E-05	RS_RS07545	*mel*; tyrosinase
RSp0803	-6.1	4.57E-05	RS_RS20955	hypothetical protein
RSp1311	-6.1	2.44E-03	RS_RS23475	hypothetical protein
RSp0572	-5.8	1.28E-04	NA	NA
RSc2348	-5.7	6.96E-05	RS_RS11785	hypothetical protein
RSc0591	-5.7	5.65E-05	RS_RS02960	hypothetical protein
RSc1706	-5.7	2.02E-03	RS_RS08620	hypothetical protein
RSp0201	-5.6	3.76E-04	RS_RS18110	decarboxylase
RSp0688	-5.5	2.15E-03	RS_RS20420	membrane protein
RSp0698	-5.5	1.41E-03	RS_RS20470	aminotransferase
RSp1601	-5.4	8.56E-04	RS_RS24795	type III effector protein
RSc2469	-5.4	3.84E-03	NA	NA
RSp1608	-5.3	1.68E-04	RS_RS24825	hypothetical protein
RSp1239	-5.3	1.81E-03	RS_RS23120	type III effector protein

RSc0228	-5.3	2.16E-03	NA	NA
RSc1801	-5.3	2.84E-03	RS_RS09090	GALA protein
RSp0724	-5.2	1.76E-04	NA	NA
RSc1502	-5.2	3.30E-04	RS_RS07550	hypothetical protein
RSp0709	-5.2	2.51E-05	RS_RS20525	cytochrome P450
RSp0770	-5.1	3.00E-03	RS_RS20805	hypothetical protein
RSc2178	-5.1	2.17E-03	RS_RS10930	hypothetical protein
RSp0735	-5.1	7.63E-04	NA	NA
RSp0175	-5.0	1.29E-04	RS_RS17985	type VI secretion protein
RSp0642	-5.0	1.64E-06	RS_RS20190	non-ribosomal peptide synthetase
RSc3251	-4.9	3.94E-05	RS_RS16280	hypothetical protein
RSp0734	-4.9	1.63E-03	RS_RS20635	hypothetical protein
RSp0817	-4.9	2.17E-03	RS_RS21025	adeC/adeK/oprM family multidrug efflux complex outer membrane factor
RSp0629	-4.9	2.64E-04	RS_RS20130	VGR-related protein
RSc1353	-4.8	7.27E-04	RS_RS06785	*ilvG*; thiamine pyrophosphate protein
RSc1756	-4.7	2.80E-05	RS_RS08870	*pehB*; exo-poly-galacturonase signal peptide protein
RSp0873	-4.7	3.88E-03	RS_RS21310	*hrpB*; regulatory protein HrpB
RSc2182	-4.7	4.92E-03	RS_RS10950	hypothetical protein
RSc1649	-4.7	6.72E-04	RS_RS08310	membrane protein
RSp0559	-4.7	3.73E-04	RS_RS19820	membrane protein
RSp0752	-4.7	9.72E-04	RS_RS20715	membrane protein
RSp1461	-4.6	2.94E-03	RS_RS24160	hypothetical protein
RSc1802	-4.6	1.34E-03	RS_RS09095	transcriptional regulator
RSc1895	-4.5	3.53E-03	RS_RS09570	helix-turn-helix transcriptional regulator
RSp1022	-4.5	3.79E-04	RS_RS22055	hypothetical protein
RSc0894	-4.5	1.41E-03	NA	NA
RSc1705	-4.5	8.92E-05	NA	NA
RSp0751	-4.4	3.00E-03	RS_RS20710	hypothetical protein
RSc0050	-4.3	7.90E-04	RS_RS00250	hypothetical protein
RSc2510	-4.3	3.32E-04	RS_RS12585	signal peptidase
RSc3252	-4.3	5.19E-05	RS_RS16285	hypothetical protein
RSp1285	-4.2	5.46E-04	RS_RS23350	*scrA*; PTS sugar transporter
RSp1240	-4.2	1.96E-04	RS_RS23125	murein transglycosylase
RSc3376	-4.2	1.61E-03	RS_RS16915	hypothetical protein
RSc3378	-4.1	2.87E-04	RS_RS16925	hypothetical protein
RSc1707	-4.1	7.62E-05	RS_RS08625	membrane protein
RSp0784	-4.1	4.20E-03	RS_RS20860	omega-3 fatty acid desaturase
RSc0972	-4.0	5.76E-04	RS_RS04810	hypothetical protein
RSp0874	-4.0	2.83E-03	RS_RS21315	*hrcC*; type III secretion system outer membrane ring protein
RSp0818	-3.8	2.77E-04	RS_RS21030	multidrug efflux RND transporter permease subunit
RSp0763	-3.8	1.62E-04	RS_RS20770	type VI secretion protein VasK

RSc2317	-3.8	4.04E-03	NA	NA
RSp0294	-3.8	4.72E-04	RS_RS18570	hemolysin-type calcium-binding protein
RSp0309	-3.8	5.26E-05	RS_RS18630	PadR family transcriptional regulator
RSp0819	-3.8	3.37E-03	RS_RS21035	hemolysin D
RSp0534	-3.7	4.12E-03	RS_RS19730	*icc*; phosphohydrolase
RSc0830	-3.7	2.63E-03	RS_RS04145	hypothetical protein
RSc3430	-3.6	4.06E-03	RS_RS17160	type VI secretion protein
RSp1575	-3.6	3.77E-04	RS_RS24695	amino acid ABC transporter substrate-binding protein
RSc0769	-3.6	7.54E-05	RS_RS03870	hydrolase
RSp1274	-3.6	2.58E-03	RS_RS23290	hypothetical protein
RSp1183	-3.6	2.59E-03	RS_RS22840	secretion protein HlyD
RSc0831	-3.6	2.79E-04	RS_RS04150	hypothetical protein
RSc1650	-3.5	1.29E-04	RS_RS08315	hypothetical protein
RSc1651	-3.5	7.97E-05	RS_RS08320	hypothetical protein
RSp0178	-3.5	4.58E-04	RS_RS18005	hypothetical protein
RSc0064	-3.4	1.77E-04	RS_RS00320	AMP-dependent synthetase
RSc2902	-3.4	1.44E-03	RS_RS14540	hypothetical protein
RSc0948	-3.4	1.86E-03	RS_RS04715	serine/threonine dehydratase
RSp1579	-3.4	2.03E-04	RS_RS24715	helix-turn-helix transcriptional regulator
RSc2923	-3.4	1.72E-03	RS_RS14640	two-component sensor histidine kinase
RSp0958	-3.3	1.01E-03	RS_RS21735	iron-sulfur cluster repair di-iron protein
RSp0219	-3.3	9.42E-04	RS_RS18205	acyl-CoA dehydrogenase
RSp1282	-3.3	4.02E-03	RS_RS23335	phosphoenolpyruvate--protein phosphotransferase
RSp1581	-3.2	3.21E-04	RS_RS24725	*katE*; catalase
RSp1555	-3.2	1.28E-04	RS_RS24595	*popF1*; hypothetical protein
RSc2470	-3.1	4.19E-04	RS_RS12400	type IV secretion protein Rhs
RSp1202	-3.1	3.86E-05	RS_RS22935	polymerase
RSp0792	-3.1	4.00E-03	RS_RS20900	fatty acid desaturase
RSp1169	-3.0	1.90E-03	RS_RS22770	metallophosphoesterase
RSp1060	-2.9	9.68E-04	RS_RS22250	membrane protein
RSp1574	-2.9	5.36E-04	RS_RS24690	transcriptional regulator
RSc3437	-2.8	4.59E-04	NA	NA
RSc2730	-2.7	1.29E-03	RS_RS13660	*pntAa*; NAD(P) transhydrogenase subunit alpha
RSp1203	-2.7	9.18E-04	RS_RS22940	diguanylate cyclase
RSc3436	-2.7	1.41E-03	NA	NA
RSc1652	-2.6	1.99E-03	RS_RS08325	*crtB*; squalene synthase HpnD
RSc0115	-2.6	1.41E-03	RS_RS00565	hemagglutinin
RSp0337	-2.5	2.03E-03	RS_RS18770	porin
RSp0876	-2.5	4.68E-04	RS_RS21325	*popB*; protein PopB
RSc1874	-2.5	4.75E-03	RS_RS09465	NUDIX hydrolase
tRNA-glyV	-2.5	4.81E-03	RS_RS05320	tRNA-Gly

RSp0113	-2.5	3.50E-03	RS_RS17695	membrane protein
RSp0112	-2.5	1.98E-03	RS_RS17690	carbonic anhydrase
RSc0337	-2.4	4.99E-04	RS_RS01660	polyphenol oxidase
RSp0855	-2.4	1.85E-03	RS_RS21220	*hrpY*; membrane protein
RSc1503	-2.4	3.92E-03	RS_RS07555	hypothetical protein
RSp0877	-2.3	8.83E-04	RS_RS21330	*popA*; protein PopA1
RSp0875	-2.3	2.64E-03	RS_RS21320	*popC*; protein PopC
RSc2824	-2.3	3.41E-03	RS_RS14125	hypothetical protein
RSp0295	-2.2	1.87E-04	RS_RS18575	hemolysin
RSc2662	-2.1	3.81E-03	RS_RS13330	mechanosensitive ion channel protein MscS
RSc0724	-2.0	3.34E-03	RS_RS03645	*pilY*; pilus biosynthesis protein PilY
RSc2734	-2.0	2.21E-03	RS_RS13680	hypothetical protein
RSc3036	-2.0	1.00E-03	RS_RS15210	*rplJ*; 50S ribosomal protein L10

[a] These are the original gene locus tags according to www.ncbi.nlm.nih.gov/nuccore/AL646052.1 and www.ncbi.nlm.nih.gov/nuccore/AL646053.1

[b] Fold-change in gene expression in Δ*phcA* compared to wild-type.

[c] False discovery rate (FDR), shown as adjusted *P*-value. FDR values comes from *P*-values corrected for multiple hypothesis testing with the Benjamini-Hochberg procedure.

[d] The new gene locus tags according to www.ncbi.nlm.nih.gov/nuccore/NC_003295.1 and www.ncbi.nlm.nih.gov/nuccore/NC_003296.1

[e] Gene annotations based on www.ncbi.nlm.nih.gov/nuccore/NC_003295.1 and www.ncbi.nlm.nih.gov/nuccore/NC_003296.1

NA indicates new locus tags and annotations are not available

Table S1B. List of putative *R. solanacearum* transporter genes differentially expressed in Δ*phcA in planta*

Putative transporter gene(s) [a]	Fold Change [b]	Annotation [c]
**Amino acids**		
RSc0481	3.1	amino-acid-binding periplasmic (PBP) ABC transporter protein
gltJ	2.5	glutamate/aspartate transmembrane ABC transporter protein
gltK	2.7	glutamate/aspartate transmembrane ABC transporter protein
gltL	3.2	putative glutamate/aspartate ATP-binding ABC transporter protein
RSp0481	3.4	substrate-binding periplasmic (PBP) ABC transporter protein
RSp1575	-3.6	amino-acid-binding periplasmic (PBP) ABC transporter protein
RSc2106	-10.7	amino-acid ATP-binding ABC transporter protein, probable *glnQ*
RSp0013	2.0	amino acid ABC transporter substrate-binding protein
RSc2257	2.6	amino-acid-binding periplasmic ABC transporter protein
RSc3386	2.3	putative outer membrane signal peptide protein, *metQ*
RSp0064	7.2	putative glycine betaine transmembrane periplasmic ABC transporter
**Branched chain amino acids**		
RSp1592	3.6	leucine-specific binding precursor transmembrane protein
RSc1751	6.4	putative amino acid transmembrane transmembrane protein
RSc1752	5.1	amino acid ABC transporter permease
RSc1753	4.2	amino-acid ATP-binding ABC transporter protein
RSc1754	5.2	amino-acid ATP-binding ABC transporter protein
RSc2244	2.2	substrate-binding periplasmic ABC transporter protein
RSc3329	2.7	amino-acid-binding periplasmic (PBP) ABC transporter protein
RSc3341	4.8	transmembrane ABC transporter protein
RSc3342	4.8	putative substrate-binding periplasmic (PBP) ABC transporter protein
RSc3343	4.6	ABC transporter ATP-binding protein
RSc3344	4.1	ABC transporter ATP-binding protein
**Glutathione**		
gsiA	2.9	ABC transporter ATP-binding protein
gsiB	3.7	substrate-binding periplasmic (PBP) ABC transporter protein
gsiC	3.3	transmembrane ABC transporter protein
gsiD	3.1	transmembrane ABC transporter protein
**Glycerol and Glycerol 3-phosphate**		
RSc3051	4.9	putative signal peptide protein
RSc3047	4.3	ABC transporter ATP-binding protein
RSc3048	3.1	sugar-phosphate transmembrane ABC transporter protein
RSc3049	2.3	transmembrane ABC transporter protein
RSc1264	3.7	glycerol-3-phosphate-binding periplasmic lipoprotein signal peptide
RSc1265	3.1	SN-glycerol-3-phosphate transmembrane ABC transporter protein
RSc1266	3.3	SN-glycerol-3-phosphate transmembrane ABC transporter protein
RSc1267	3.4	glycerol-3-phosphate transporter ATP-binding subunit

**Arabinose**		
RSc2758	-23.9	L-arabinose ABC transporter periplasmic-binding protein
RSc2756	-9.0	L-arabinose transporter permease protein
RSc2757	-13.6	L-arabinose transporter ATP-binding protein
**Myo-inositol**		
RSc1241	4.9	putative rhizopine-binding precursor signal peptide protein
RSc1243	3.7	sugar transmembrane ABC transporter protein
RSc1242	4.3	sugar ATP-binding ABC transporter protein
**Dicarboxylates**		
RSp1614	4.0	transmembrane protein, probable MFS type D-galactonate transporter
RSp1396	3.7	transmembrane protein, probable MFS type D-galactonate transporter
gudP	8.2	putative glucarate transporter (D-glucarate permease)
RSc3059	3.4	involved in the uptake of citrate and transport of protons into the cell
**Polyamines**		
RSc1734	8.5	putative Fe3+/spermidine/putrescine ABC transporter
RSc1732	5.3	putative polyamine ABC transporter substrate-binding protein
RSc1731	3.1	putative polyamine ABC transporter permease
RSc1733	13.9	putative spermidine/putrescine ABC transporter
**Nitrate**		
narK1	5.2	nitrite/nitrate ABC transporter transmembrane protein
narK2	4.8	nitrite/nitrate ABC transporter transmembrane protein
**Iron**		
RSc2937	22.5	putative periplasmic iron-binding signal peptide protein
RSc2938	20.9	putative transmembrane ABC transporter protein
RSc2939	15.6	ABC transporter ATP-binding protein
RSp0243	10.0	heme ABC transporter
**Others**		
RSc1340	3.6	aliphatic sulfonate ABC transporter transmembrane protein
RSp1595	7.9	3-(3-hydroxy-phenyl) propionate transporter MhpT
RSp0638	-9.0	ABC transporter ATP-binding protein
RSp1458	4.3	transmembrane protein
RSp0726	-14.9	MFS (Major facilitator superfamily) transporter
RSp0499	-9.4	MFS transporter
RSp1047	3.0	MFS transporter
RSc0547	5.7	transport of small molecules; carbohydrates, organic acids, alcohols
RSc1975	2.5	putative transmembrane ABC transporter protein, lipooligosaccharide
RSc2041	2.2	putative urea/short-chain amide-binding signal peptide protein
RSc1080	6.1	putative hexuronate transporter transmembrane protein

[a] Transporter function and substrate identification are based on annotation (www.ncbi.nlm.nih.gov/nuccore/NC_003295.1 and www.ncbi.nlm.nih.gov/nuccore/NC_003296.1); and manual curation for binding sites or other identifying motifs. For full GenBank locus tags, see Table S1.

[b] These genes are differentially regulated in the Δ*phcA* mutant relative to wild-type at an FDR <0.005.

[c] Gene annotations are from www.ncbi.nlm.nih.gov/nuccore/NC_003295.1 and www.ncbi.nlm.nih.gov/nuccore/NC_003296.1

Table S1C. **List of putative *R. solanacearum* adhesion genes differentially expressed in Δ*phcA in planta***

Gene [a]	Fold change [b]	Annotation [c]
RSp1605	-12.0	hemagglutinin-like protein
RSp1180	-7.1	putative hemagglutinin/hemolysin-related protein
RSc0115	-2.6	putative hemagglutinin-related protein
RSc2796	14.1	putative hemagglutinin-related transmembrane protein
RSc2797	9.4	putative hemagglutinin-related transmembrane protein
RSp1620	4.9	putative hemagglutinin-related protein

[a] The original locus tag for genes according to www.ncbi.nlm.nih.gov/nuccore/AL646052.1 and www.ncbi.nlm.nih.gov/nuccore/AL646053.1

[b] These genes are differentially regulated in the Δ*phcA* mutant relative to wild-type at an FDR <0.005

[c] Gene annotations are from www.ncbi.nlm.nih.gov/nuccore/NC_003295.1 and www.ncbi.nlm.nih.gov/nuccore/NC_003296.1
