# Supplementary figures and images for: A Single Regulator Mediates Strategic Switching between Attachment/Spread and Growth/Virulence in the Plant Pathogen Ralstonia solanacearum
Source: mBio. 2017 Sep 26;8(5):e00895-17. doi: 10.1128/mBio.00895-17 (PMC5615195; doi:10.1128/mBio.00895-17)

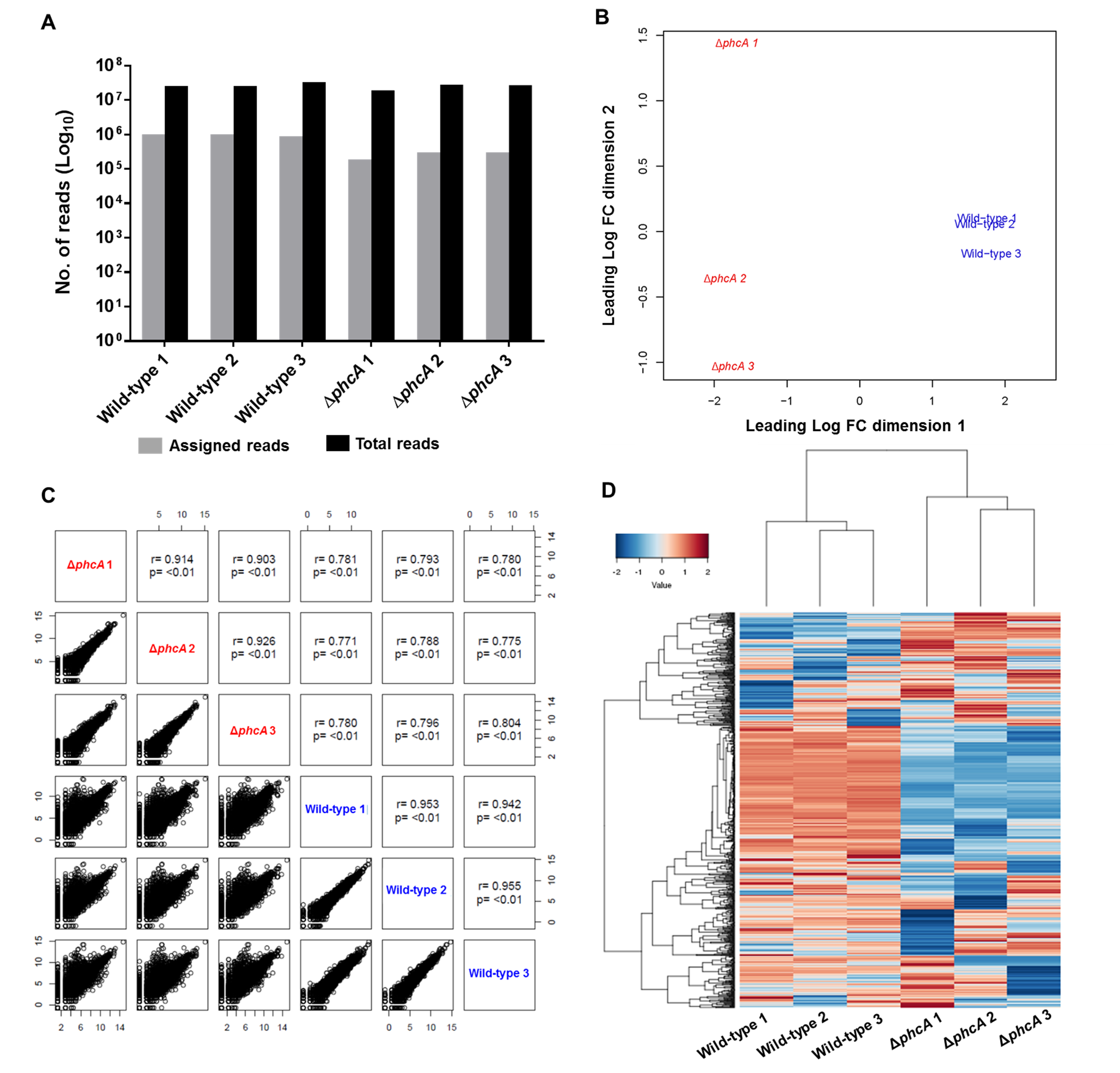

Supplement: FIG S1 [file mbo005173501sf1.tif]

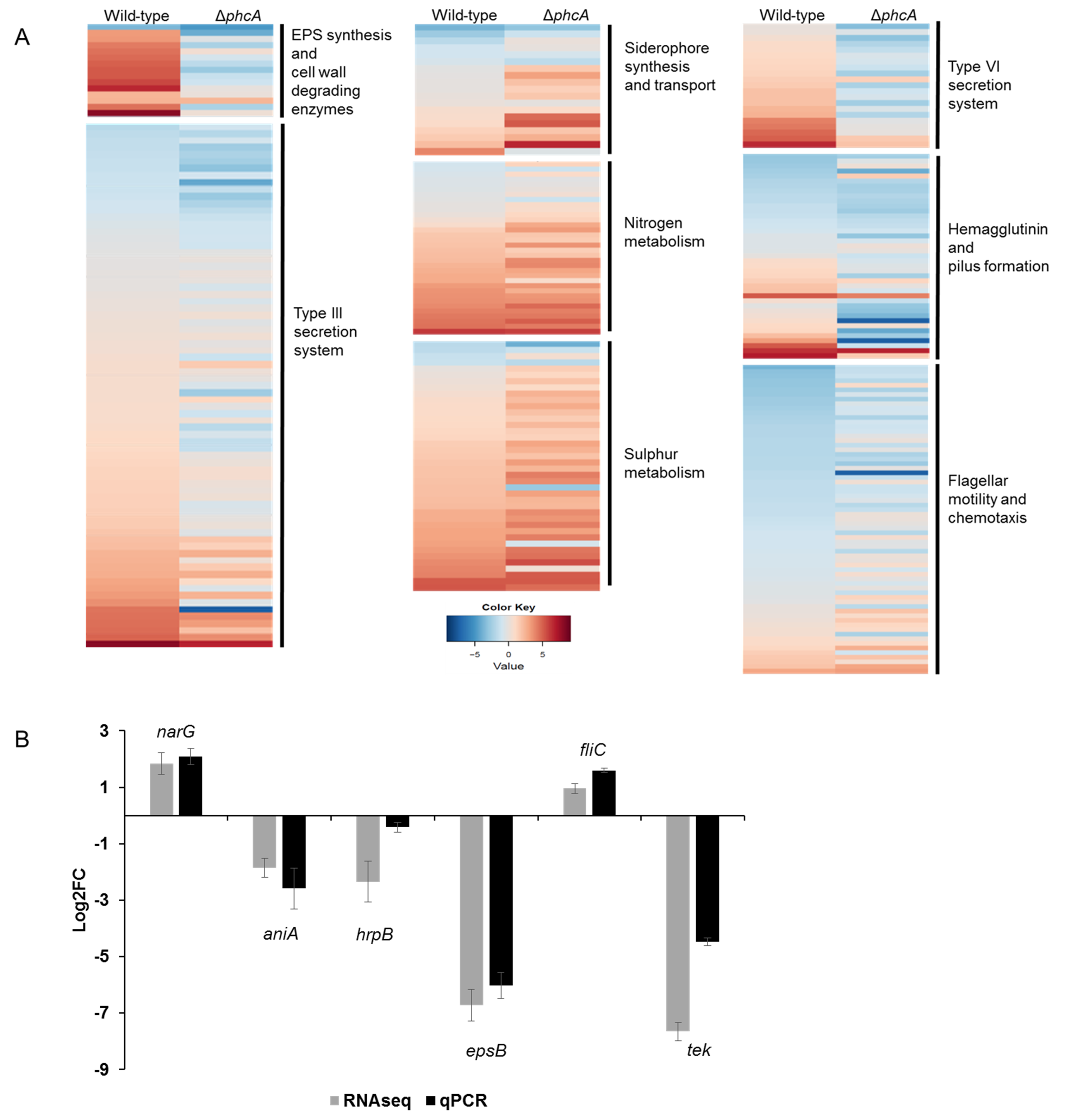

Supplement: FIG S2 [file mbo005173501sf2.tif]

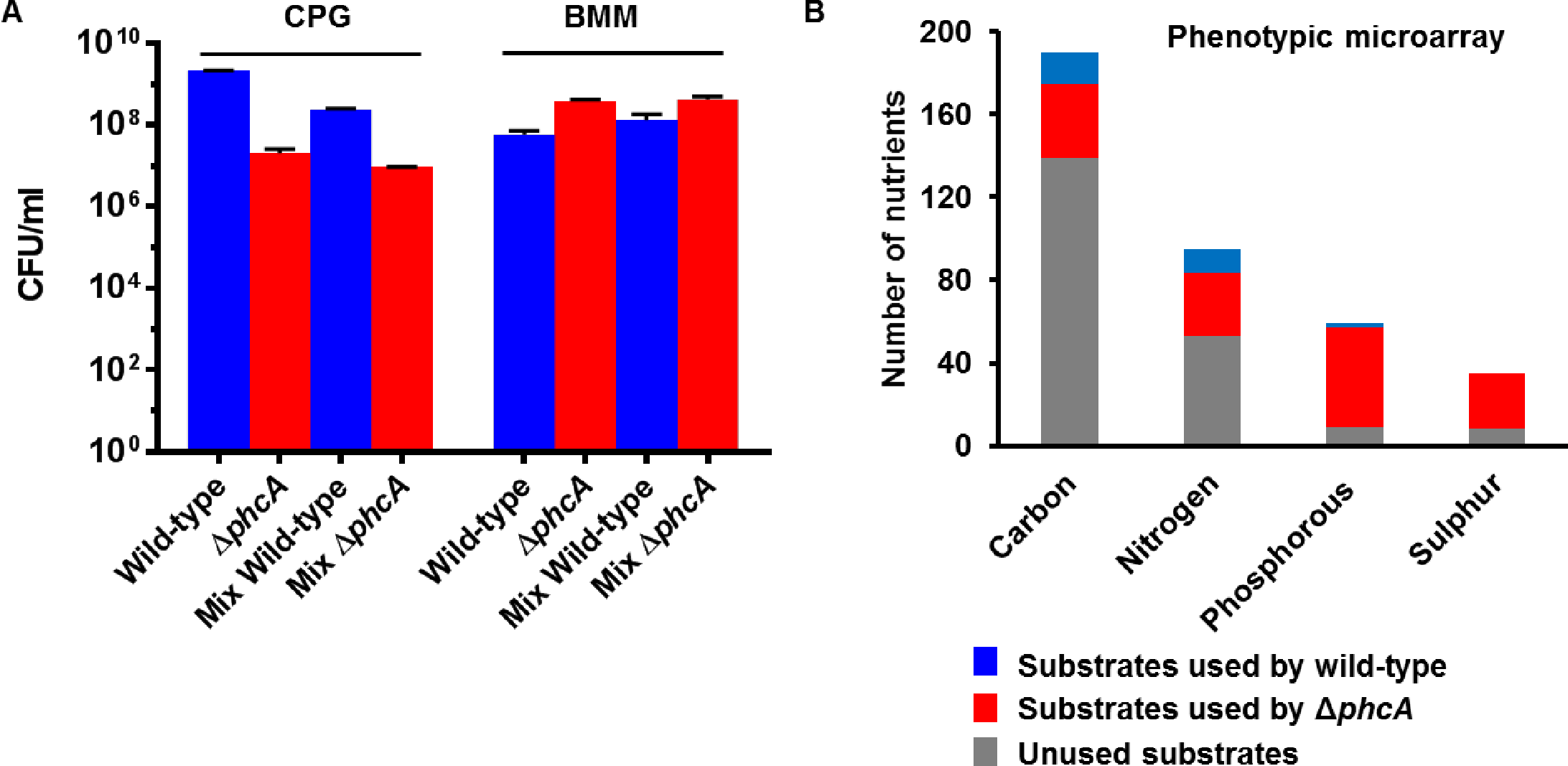

Supplement: FIG S3 [file mbo005173501sf3.tif]

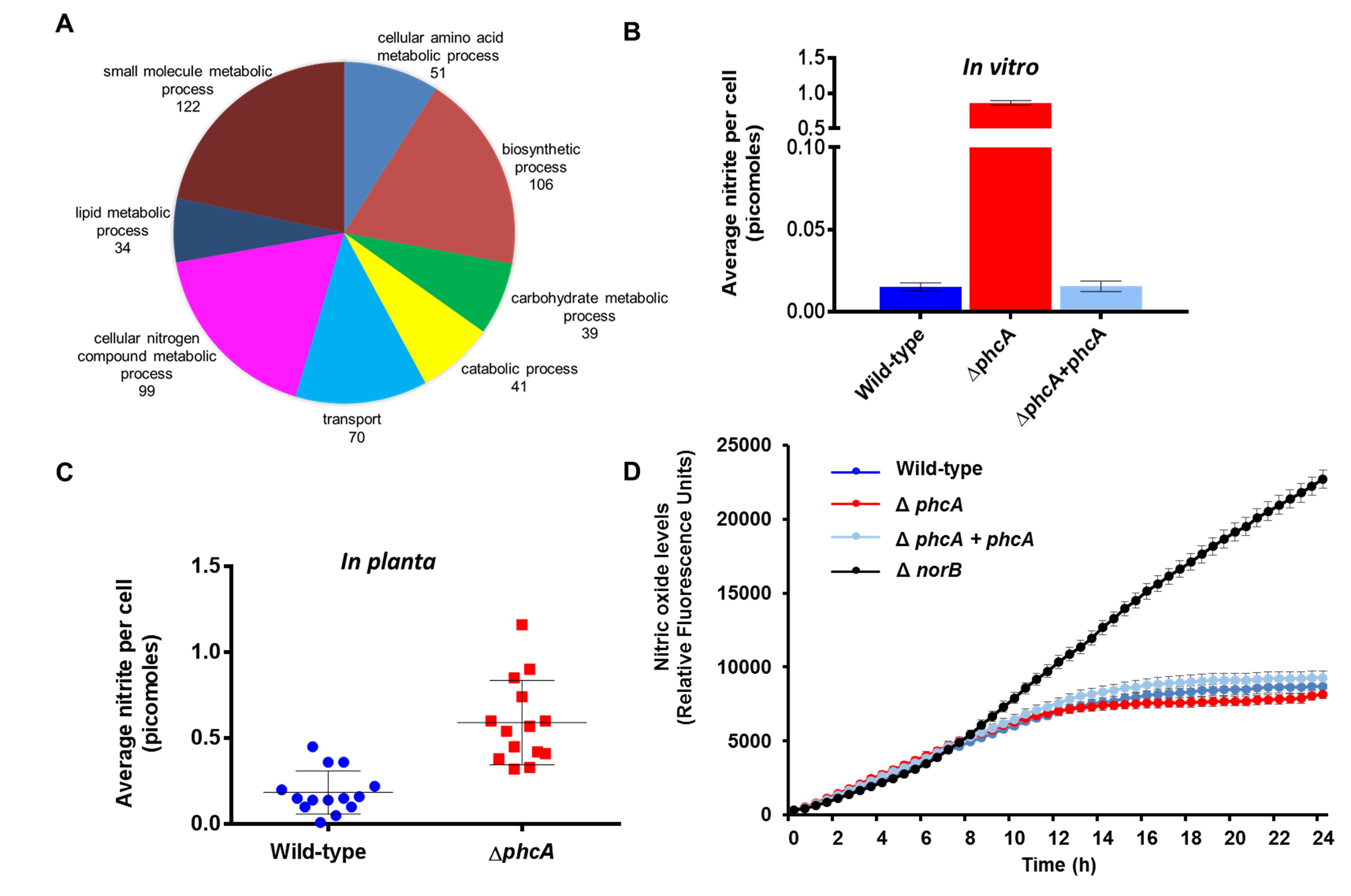

Supplement: FIG S4 [file mbo005173501sf4.tif]

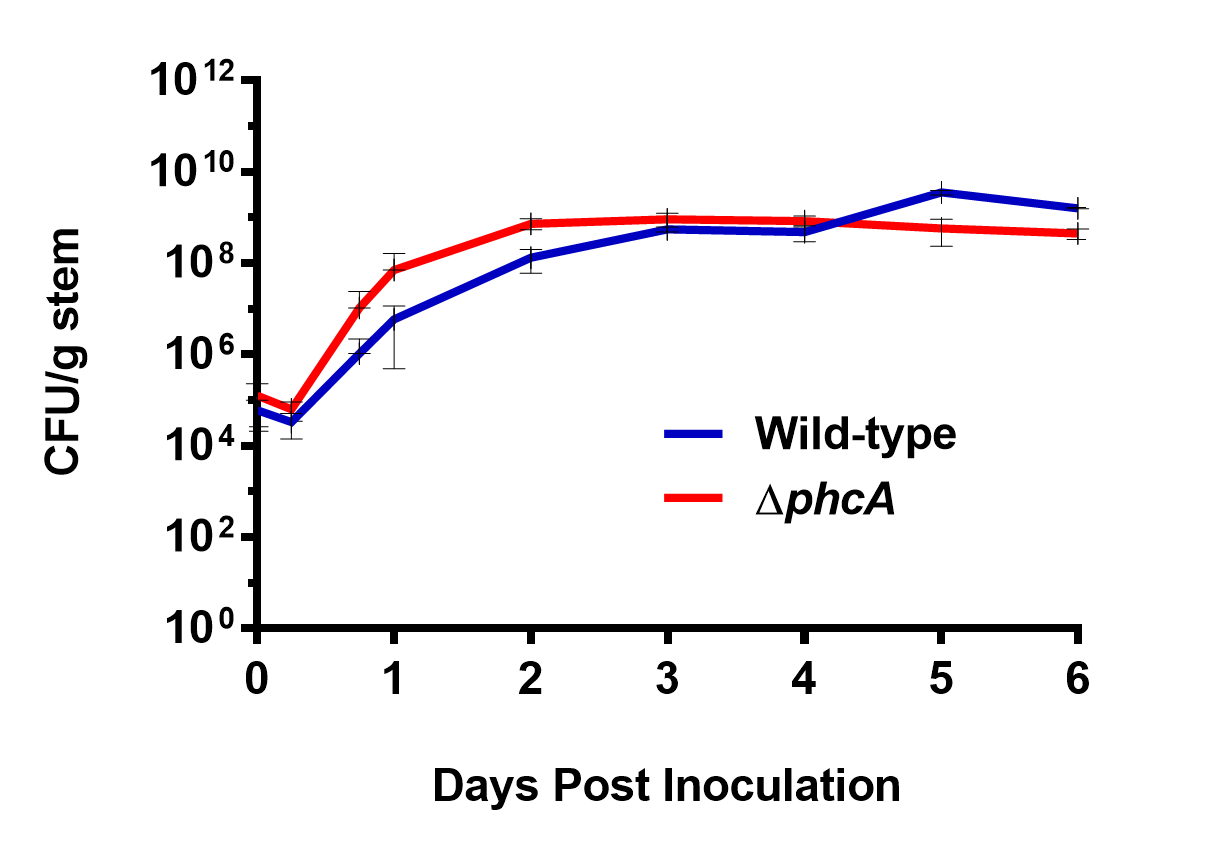

Supplement: FIG S5 [file mbo005173501sf5.tif]
