# Supplementary material for: A Single Regulator Mediates Strategic Switching between Attachment/Spread and Growth/Virulence in the Plant Pathogen Ralstonia solanacearum
Source: mBio. 2017 Sep 26;8(5):e00895-17. doi: 10.1128/mBio.00895-17 (PMC5615195; doi:10.1128/mBio.00895-17)
Supplement: TABLE S2 [file mbo005173501st2.pdf]

Table S2. Growth phenotypes gained by *R. solanacearum* Δ*phcA* on Biolog plate substrates

Plate Type [a]	Wells [b]	Test [c]	Area [d]	Nutrient(s) [e]
PM01	A07	L-Aspartic Acid *	40090.5	C-Source, amino acid
PM01	A08	L-Proline *	31937.5	C-Source, amino acid
PM01	B12	L-Glutamic Acid *	32384	C-Source, amino acid
PM01	D01	L-Asparagine *	18324.5	C-Source, amino acid
PM01	E01	L-Glutamine *	25325	C-Source, amino acid
PM01	G03	L-Serine *	34111	C-Source, amino acid
PM01	G04	L-Threonine *	26109	C-Source, amino acid
PM01	G05	L-Alanine *	31224.5	C-Source, amino acid
PM02	G06	L-Histidine *	45897	C-Source, amino acid
PM02	H03	L-Pyroglutamic Acid	25456	C-Source, amino acid
PM01	A10	D-Trehalose *	26467	C-Source, carbohydrate
PM01	A12	Dulcitol (galactitol) *	31256	C-Source, carbohydrate
PM01	C09	a-D-Glucose *	46229.5	C-Source, carbohydrate
PM01	F03	m-Inositol *	27059	C-Source, carbohydrate
PM01	A04	D-Saccharic Acid (Glucaric acid) *	58272	C-Source, carboxylic acid
PM01	A05	Succinic Acid *	54940.5	C-Source, carboxylic acid
PM01	B05	D-Glucuronic Acid	37024	C-Source, carboxylic acid
PM01	B06	D-Gluconic Acid *	39678	C-Source, carboxylic acid
PM01	C03	D,L-Malic Acid *	44288	C-Source, carboxylic acid
PM01	F02	Citric Acid *	54313	C-Source, carboxylic acid
PM01	F05	Fumaric Acid *	32817	C-Source, carboxylic acid
PM01	F08	Mucic Acid (galactaric acid) *	57037	C-Source, carboxylic acid
PM01	G12	L-Malic Acid *	39762	C-Source, carboxylic acid
PM01	H08	Pyruvic Acid *	27990.5	C-Source, carboxylic acid
PM01	H09	L-Galactonic Acid-g-Lactone	58369.5	C-Source, carboxylic acid
PM01	H10	D-Galacturonic Acid	57629	C-Source, carboxylic acid
PM02	D10	g-Amino-N-Butyric Acid *	29857.5	C-Source, carboxylic acid
PM02	D12	Butyric Acid	15968.5	C-Source, carboxylic acid
PM02	E08	b-Hydroxybutyric Acid *	27990	C-Source, carboxylic acid
PM02	F06	Quinic Acid *	46145.5	C-Source, carboxylic acid
PM02	F12	L-Tartaric Acid	18772.5	C-Source, carboxylic acid
PM01	G10	Methylpyruvate *	25134.5	C-Source, ester
PM03	B02	Glycine *	19789.5	N-Source, amino acid
PM03	B03	L-Histidine *	40466.5	N-Source, amino acid
PM03	B09	L-Proline *	22851	N-Source, amino acid
PM03	B10	L-Serine *	33471.5	N-Source, amino acid
PM03	B11	L-Threonine *	28624	N-Source, amino acid
PM03	B12	L-Tryptophan	9299.5	N-Source, amino acid
PM03	C03	D-Alanine *	21488.5	N-Source, amino acid
PM03	C05	D-Aspartic Acid	39935.5	N-Source, amino acid
PM03	C06	D-Glutamic Acid	19898.5	N-Source, amino acid
PM03	C10	L-Citrulline *	30283.5	N-Source, amino acid
PM03	D03	L-Pyroglutamic Acid	25276	N-Source, amino acid
PM03	A02	Ammonia	49407.5	N-Source, inorganic
PM03	A03	Nitrite	31001	N-Source, inorganic
PM03	A04	Nitrate *	48364	N-Source, inorganic

PM03	A05	Urea *	45439.5	N-Source, other
PM03	F06	Guanine	43857.5	N-Source, other
PM03	F07	Guanosine	36200.5	N-Source, other
PM03	F12	Inosine	24070.5	N-Source, other
PM03	G01	Xanthine	30011	N-Source, other
PM03	G03	Uric Acid	35955.5	N-Source, other
PM03	G08	g-Amino-N-Butyric Acid *	39482	N-Source, other
PM03	H03	Ala-Glu	28185.5	N-Source, peptide
PM04	A02	Phosphate	56590	P-Source, inorganic
PM04	A03	Pyrophosphate	56971.5	P-Source, inorganic
PM04	A04	Trimetaphosphate	46706.5	P-Source, inorganic
PM04	A05	Tripolyphosphate	53166.5	P-Source, inorganic
PM04	B01	Thiophosphate	43514	P-Source, inorganic
PM04	B02	Dithiophosphate	46506.5	P-Source, inorganic
PM04	A08	Adenosine 2`-Monophosphate	50960.5	P-Source, organic
PM04	A09	Adenosine 3`-Monophosphate	46908.5	P-Source, organic
PM04	A10	Adenosine 5`-Monophosphate	49712	P-Source, organic
PM04	A11	Adenosine 2`,3`-Cyclic Monophosphate	43255	P-Source, organic
PM04	B03	D,L-a-Glycerol Phosphate	50165.5	P-Source, organic
PM04	B04	b-Glycerol Phosphate	48360.5	P-Source, organic
PM04	B06	D-2-Phospho-Glyceric Acid	54472.5	P-Source, organic
PM04	B07	D-3-Phospho-Glyceric Acid	56664	P-Source, organic
PM04	B08	Guanosine 2`-Monophosphate	45278.5	P-Source, organic
PM04	B09	Guanosine 3`-Monophosphate	46667	P-Source, organic
PM04	B10	Guanosine 5`-Monophosphate	48251.5	P-Source, organic
PM04	C01	Phosphoenol Pyruvate	47397.5	P-Source, organic
PM04	C02	Phospho-Glycolic Acid	45471.5	P-Source, organic
PM04	C03	D-Glucose-1-Phosphate *	48236.5	P-Source, organic
PM04	C04	D-Glucose-6-Phosphate	44694.5	P-Source, organic
PM04	C05	2-Deoxy-D-Glucose 6-Phosphate	50699	P-Source, organic
PM04	C06	D-Glucosamine-6-Phosphate	48652	P-Source, organic
PM04	C07	6-Phospho-Gluconic Acid	55409.5	P-Source, organic
PM04	C08	Cytidine 2`-Monophosphate	51350	P-Source, organic
PM04	C09	Cytidine 3`-Monophosphate	46718.5	P-Source, organic
PM04	C10	Cytidine 5`-Monophosphate	45070.5	P-Source, organic
PM04	C11	Cytidine 2`,3`-Cyclic Monophosphate	37698.5	P-Source, organic
PM04	C12	Cytidine 3`,5`-Cyclic Monophosphate	25363	P-Source, organic
PM04	D01	D-Mannose-1-Phosphate	40951	P-Source, organic
PM04	D02	D-Mannose-6-Phosphate	42695.5	P-Source, organic
PM04	D03	Cysteamine-S-Phosphate	46915.5	P-Source, organic
PM04	D04	Phospho-L-Arginine	49426.5	P-Source, organic
PM04	D05	O-Phospho-D-Serine	51723	P-Source, organic
PM04	D06	O-Phospho-L-Serine	53257	P-Source, organic
PM04	D07	O-Phospho-L-Threonine	50154	P-Source, organic

PM04	D08	Uridine 2`-Monophosphate	49059	P-Source, organic
PM04	D09	Uridine 3`-Monophosphate	50122	P-Source, organic
PM04	D10	Uridine 5`-Monophosphate	47772.5	P-Source, organic
PM04	D11	Uridine 2`,3`-Cyclic Monophosphate	43864	P-Source, organic
PM04	E01	O-Phospho-D-Tyrosine	42393	P-Source, organic
PM04	E02	O-Phospho-L-Tyrosine	39619.5	P-Source, organic
PM04	E03	Phosphocreatine	50696.5	P-Source, organic
PM04	E04	Phosphoryl Choline	44046.5	P-Source, organic
PM04	E05	O-Phosphoryl-Ethanolamine	48115	P-Source, organic
PM04	E09	Thymidine 3`-Monophosphate	48093.5	P-Source, organic
PM04	E10	Thymidine 5`-Monophosphate	49231.5	P-Source, organic
PM04	E11	Inositol Hexaphosphate	40832.5	P-Source, organic
PM04	F02	Sulfate	49470.5	S-Source, inorganic
PM04	F03	Thiosulfate	47178	S-Source, inorganic
PM04	F04	Tetrathionate	46468	S-Source, inorganic
PM04	F05	Thiophosphate	45956	S-Source, inorganic
PM04	F06	Dithiophosphate	54899.5	S-Source, organic
PM04	F07	L-Cysteine	54087.5	S-Source, organic
PM04	F08	D-Cysteine	50186	S-Source, organic
PM04	F09	Cys-Gly	50247.5	S-Source, organic
PM04	F10	L-Cysteic Acid	49497	S-Source, organic
PM04	F11	Cysteamine	37190.5	S-Source, organic
PM04	F12	L-Cysteine Sulfinic Acid	47504	S-Source, organic
PM04	G02	S-Methyl-L-Cysteine	36078	S-Source, organic
PM04	G03	Cystathionine	43588	S-Source, organic
PM04	G04	Lanthionine	42943	S-Source, organic
PM04	G05	Glutathione	37641.5	S-Source, organic
PM04	G07	L-Methionine *	53752	S-Source, organic
PM04	G08	D-Methionine	50138.5	S-Source, organic
PM04	G09	Gly-Met	53437	S-Source, organic
PM04	G10	N-Acetyl-D,L-Methionine	48371	S-Source, organic
PM04	G11	L-Methionine Sulfoxide	48806	S-Source, organic
PM04	G12	L-Methionine Sulfone	39562.5	S-Source, organic
PM04	H01	L-Djenkolic Acid	39412	S-Source, organic
PM04	H02	Thiourea	33875	S-Source, organic
PM04	H05	Taurocholic Acid	36527.5	S-Source, organic
PM04	H06	Taurine *	48386.5	S-Source, organic
PM04	H07	Hypotaurine	50062.5	S-Source, organic
PM04	H09	Butane Sulfonic Acid	51184.5	S-Source, organic
PM04	H10	2-Hydroxyethane Sulfonic Acid	48638	S-Source, organic
PM04	H11	Methane Sulfonic Acid	49897.5	S-Source, organic

[a] Biolog plate type.
[b] Well location for this substrate in the corresponding Biolog plate type.
[c] Substrates for which Δ*phcA* gained a growth phenotype when compared to wild-type.
[d] Area was measured as the sum of reads during bacterial growth over 72 h ($P < 0.01$).

[e] Substrate or nutrient tested.
* Asterisk indicates nutrients found in tomato xylem sap.
