# Supplementary material for: A Single Regulator Mediates Strategic Switching between Attachment/Spread and Growth/Virulence in the Plant Pathogen Ralstonia solanacearum
Source: mBio. 2017 Sep 26;8(5):e00895-17. doi: 10.1128/mBio.00895-17 (PMC5615195; doi:10.1128/mBio.00895-17)
Supplement: TABLE S3 [file mbo005173501st3.pdf]

Table S3. Biochemistry of the xylem habitat occupied by *R. solanacearum*

**Compounds present in tomato xylem sap** [a]	**Growth (C or N source)** [b]	**Pathway Function** [c]	**Kegg Path-way** [d]	**Gene** [e]	**FC** [f]	**Annotation** [g]
**Sugar**						
Unknown disaccharide	NT			RSc1081	7.1	glucosidase
sucrose [1,2,,] •	Both	Sucrose utilization	rso00500	RSp1285 (*scrA*)	-4.2	PTS system, sucrose-specific (IIBC component) transmembrane protein
trehalose •	Wild-type	Trehalose utilization	rso00500	RSp0277 (*treA*)	-7.5	periplasmic alpha,alpha-trehalase
glucose-1-phosphate •	Δ*phcA* *					
cellobiose •	Neither					
beta-gentiobiose •	Neither					
maltose •	Neither					
N-acetyl-D-hexosamine •	Neither					
digitoxose •	NT					
ribose •	Neither					
xylulose •	NT					
m-inositol •	Δ*phcA* *	Inositol utilization	rso00562	RSc1238 (*iolE*)	3.1	putative myo-inositol catabolism protein
				RSc1239 (*iolD*)	5.0	putative acetolactate synthase protein
				RSc1240 (*iolC*)	3.6	transferase kinase protein
				RSc1244 (*iolH*)	4.5	putative protein in m-inositol catabolic pathway
				RSc1245 (*iolI*)	5.0	putative protein in m-inositol catabolic pathway
				RSc1246 (*iolG*)	4.2	putative oxidoreductase m-inositol 2-dehydrogenase
				RSc1247	4.1	putative myo-inositol catabolism protein
erythrose •	NT					
galactinol •	NT					
glycerol-3-galactoside •	NT					
hexitol (mannitol) •	Both					
fucose •	Neither					
N-acetylmannosamine •	Neither					
1-methylgalactose •	NT					
6-deoxyglucose •	NT					
xylose •	Neither					
levoglucosan •	NT					
3,6-anhydro-D-galactose •	NT					
fructose [2,] •	Both					
glucose [2,] •	Δ*phcA* *			RSc1231 (*tal*)	2.1	transaldolase
galactose [2,] •	Wild-type	Galactose utilization	rso00052	RSc2751 (*dgoAb*)	-15.0	putative galactonate dehydratase protein
				RSc2759	-13.8	2-dehydro-3-deoxygalactonokinase protein

				(*dgoK*)		
mannose [2, •]	Neither					
**Amino acids**						
arginine [2,3,4]	Both					
tyrosine [2,3,4, •]	Both					
phenylalanine [2,3,4, •]	Both	Phenyl-alanine synthesis	rso00400	RSc2512	2.2	arogenate dehydratase
isoleucine [2,3,4, •]	Both	Valine, isoleucine, and leucine degradation	rso00280	RSc1762	3.8	acyl-CoA dehydrogenase
valine [2,3,4, •]	Δ*phcA*			RSc2020	4.6	acyl-CoA dehydrogenase
leucine [2,3,4, •]	Δ*phcA*			RSc0475	2.6	acetyl-CoA acetyltransferase
				RSp1591	3.0	aldehyde dehydrogenase
beta-alanine [•]	Both	Beta-alanine utilization	rso00410	RSc0029 (*goaG*)	7.0	4-aminobutyrate transaminase
asparagine [2,4, •]	Δ*phcA* *					
glutamine [2,4, •]	Δ*phcA* *					
aspartic acid [2,3,4]	Δ*phcA* *	Aspartate degradation		RSp1263	-32	L-aspartate oxidase
histidine [2,3,4]	Δ*phcA* *	Histidine degradation	rso00340	RSc2647	2.7	urocanate hydratase
threonine [3,4, •]	Δ*phcA* *	Isoleucine synthesis from threonine	rso00290	RSc0948	-3.4	serine/threonine dehydratase
alanine [2,3,4, •]	Δ*phcA* *					
proline [2,3,4, •]	Δ*phcA*	Proline and Ornithine degradation	rso00330	RSc0159	3.9	ornithine--oxo-acid transaminase
ornithine [2,3, •]	Neither			RSp0418	3.8	2,3-diaminopropionate biosynthesis protein SbnB
				RSp0483	2.6	ornithine cyclodeaminase
glutamic acid [2,3,4, •]	Δ*phcA* *					
methionine [3,4,5]	Δ*phcA*	Methionine synthesis	rso00270	RSc1562	2.8	O-acetylhomoserine aminocarboxypropyltransferase
				RSp0676 (*metE*)	2.2	5-methyltetrahydropteroyltriglutamate-homocysteine methyltransferase
				RSp0781	-22.4	cystathionine gamma-synthase
glycine [3, •]	Δ*phcA*	Glycine/Serine utilization	rso00260	RSp0055	9.3	serine hydroxymethyltransferase
citrulline [3, •]	Δ*phcA*					
serine [2,3, •]	Δ*phcA* *	Cysteine synthesis from serine	rso00270			
O-acetylserine [•]	Δ*phcA* *			RSp1439 (*cysE1*)	-13.0	serine acetyltransferase
Taurine [2]	Δ*phcA*	Sulfur metabolism	rso00920	RSc0746 (*tauD*)	2.8	taurine dioxygenase
lysine [2,3,4, •]	Neither	Lysine synthesis	rso00300	RSp0424	3.6	diaminopimelate decarboxylase
homoserine [•]	Neither					

N-methylalanine •	NT					
oxoproline •	NT					
hydroxylysine [2]	NT					
**Carboxylic acids**						
citric acid [4,] •	Δ*phcA* *					
4-aminobutyric acid [2,4,] •	Δ*phcA* *	GABA utilization	rso00250	RSc0028 (*gabD1*)	3.7	NAD-dependent succinate-semialdehyde dehydrogenase
				RSc0029 (*goaG*)	7.0	4-aminobutyrate transaminase
fumaric acid [4,5,] •	Δ*phcA* *					
succinic acid [4,5,] •	Δ*phcA*					
malic acid [4,5,] •	Δ*phcA* *	TCA and glyoxylate cycles	rso00020	RSp0814 (*mqo*)	4.4	malate:quinone oxidoreductase
gluconic acid •	Δ*phcA*			RSc1763	3.2	gluconate 2-dehydrogenase
pyruvic acid •	Δ*phcA* *	Valine synthesis from pyurvate	rso00290	RSc1353 (*ilvG*)	-4.8	acetolactate synthase 2 catalytic subunit
		Pyruvate metabolism	rso00620	RSc1798	4.8	2-oxoisovalerate dehydrogenase subunit beta
				RSc1799	7.4	branched-chain alpha-keto acid dehydrogenase subunit E2
galactaric acid •	Δ*phcA* *	Pectin/Galacturonate/Galactarate/glucarate utilization	rso00053	RSp0826	7.6	5-dehydro-4-deoxyglucarate dehydratase
glucaric acid •	Δ*phcA* *			RSp0827	5.3	2,5-dioxovalerate dehydrogenase
				RSp0829 (*gudD2*)	7.9	glucarate dehydratase
				RSp0830 (*garD*)	5.9	galactarate dehydratase
				RSp0831	6.3	NAD-dependent dehydratase
				RSp0832	4.9	gluconolactonase
				RSp0880*	2.2	polygalacturonase
				RSc1756 (*pehB*)	-4.7	exo-poly-galacturonase
glycolic acid •	Δ*phcA* *					
quinic acid •	Δ*phcA* *					
3-hydroxybutyric acid •	Δ*phcA* *					
glucuronic acid •	Δ*phcA* *					
ascorbic acid [5]	Neither					
2,4-diaminobutyric acid •	Neither					
lactic acid •	Neither					
benzoic acid •	Neither					
shikimic acid •	Neither					
malonic acid [5]	NT					
maleic acid [5]	NT					

threonic acid •	NT					
3-aminoisobutyric acid •	NT					
pipecolinic acid •	NT					
galactonic acid •	NT					
nonoic acid •	NT					
3-hydroxypropionic acid •	NT					
ribonic acid •	NT					
2-hydroxyvaleric acid •	NT					
hexonic acid •	NT					
adipic acid •	NT					
hydroxycarbamate •	NT					
**Amines**						
putrescine •	Neither					
ethanolamine [3,] •	Neither			RSc3127	6.9	ethanolamine ammonia lyase large subunit
phenylethylamine •	Neither					
tyramine •	Neither					
spermidine •	Neither					
phenylethylamine •	NT					
noradrenaline •	NT					
cyclohexylamine •	NT					
**Other nitrogenous compounds**						
ammonia [4]	Δ*phcA* *					
nitrate [4,6]	Δ*phcA* *	Denitri-fication	rsp00910	RSp0974 (*narG*)	3.63	respiratory nitrate reductase alpha chain oxidoreductase
				RSp0975 (*narH*)	3.28	respiratory nitrate reductase oxidoreductase
				RSp0976 (*narJ*)	4.12	respiratory nitrate reductase oxidoreductase
				RSp0977 (*narI*)	3.25	respiratory nitrate reductase transmembrane
				RSp1503 (*aniA*)	-3.5	nitrate reductase membrane protein
urea [2,] •	Δ*phcA* *					
hydroxylamine •	Neither					
thymine •	Neither					
uracil •	Neither					
adenosine •	Neither					
**Fatty acids**		Fatty Acid degradation	rso00071			
decanoic acid •	NT			RSc0064	-3.4	AMP-dependent synthetase
pentadecanoic acid •	NT			RSc1761	3.3	acetyl-CoA acetyltransferase
palmitic acid •	NT			RSc1762	3.8	acyl-CoA dehydrogenase
arachidic acid •	NT			RSc2020	4.6	acyl-CoA dehydrogenase

lauric acid •	NT			RSc0474	2.7	3-hydroxyacyl-CoA dehydrogenase
stearic acid •	NT			RSc0475	2.6	acetyl-CoA acetyltransferase
**Alcohols**						
galactitol (dulcitol) •	Δ*phcA*					
dihydroxyacetone •	Neither					
lyxitol •	Neither					
xylitol •	Neither					
ribitol •	Neither					
diacetone alcohol •	NT					
octadecanol •	NT					
pentitol •	NT					
1-hexadecanol •	NT					
threitol •	NT					
benzylalcohol •	NT					
octanol •	NT					
1-deoxyerythritol •	NT					
deoxypentitol •	NT					
**Others**						
butyrolactam •	NT					
conduritol-beta-expoxide •	NT					
piperidone •	NT					
acetophenone •	NT					
salicylaldehyde •	Neither					
glyceric acid •	NT					
1,2-dihydroxycylohexane •	NT					
propane-1,3-diol •	NT					
dodecanoic acid, isopropanol ester •	NT					
Phosphorserine •	NT					
**Compounds not known to be present in xylem sap [h]**						
Hydroxycinnamic acids	NT	Hydroxycinnamic acid degradation	Lowe et al, 2015	RSp0227 (*fcs*)<br>RSp0225 (*fca*)<br>RSp0226 (*vdh*)<br>RSc1442 (*pcaG*)<br>RSc2253 (*pcaJ*)<br>RSc2254 (*pcaI*)		

Glutathione	Δ*phcA*	Glutathione metabolism	rso00480	RSc2012 (*gstH*) RSp0274 (*ggt1*) RSp0532
Cysteine	Δ*phcA*	Sulfur Assimilation /Cysteine Synthesis	rso00920	RSc2421 (*cysN*) RSc2422 (*cysD*) RSc2425 (*cysI1*) RSp0417 (*cysK2*)

[a] Metabolites found in xylem sap are either previously known (indicated by reference number) or identified in this study (indicated by solid black circle).
[b] Growth of wild-type or Δ*phcA* bacteria in the respective metabolite, determined either by growth on the relevant Biolog plate or in Boucher's minimal medium (BMM) supplemented with 10mM metabolite as described in Materials and Methods. Asterisk indicates that Δ*phcA* grew better than wild-type in this metabolites.
[c] Pathway assignment based on KEGG.
[d] Pathway annotation number based on KEGG.
[e] Differentially expressed genes that correspond to the respective metabolite. These genes are identified by the original locus tag based on www.ncbi.nlm.nih.gov/nuccore/AL646052.1 and www.ncbi.nlm.nih.gov/nuccore/AL646053.1. Genes with green locus tags are induced at high cell density and genes with purple locus tags are induced at low cell density
[f] FC indicates gene expression fold change values in Δ*phcA* relative to wild-type.
[g] Gene annotations based on www.ncbi.nlm.nih.gov/nuccore/NC_003295.1 and www.ncbi.nlm.nih.gov/nuccore/NC_003296.1.
[h] These compounds were not detected in xylem sap, but the corresponding genes are differentially expressed in Δ*phcA* and they have been tested as growth substrates for WT and Δ*phcA Rs*.

References

1. **Jacobs JM, Babujee L, Meng F, Milling A, Allen C.** 2012. The in planta transcriptome of Ralstonia solanacearum: conserved physiological and virulence strategies during bacterial wilt of tomato. MBio **3**.
2. **Zuluaga AP, Puigvert M, Valls M.** 2013. Novel plant inputs influencing Ralstonia solanacearum during infection. Front Microbiol **4:**349.
3. **Dixon GR, Pegg GF.** 1972. Changes in amino-acid content of tomato xylem sap following infection with strains of *Verticillium albo-atrum*. Ann Bot **36:**147-154.
4. **Bialczyk J, Lechowski Z, Dziga D.** 2004. Composition of the xylem sap of tomato seedlings cultivated on media with HCO3− and nitrogen source as NO3− or NH4+. Plant and Soil **263:**1265-1272.
5. **Senden MHMN, Van Der Meer AJGM, Limborgh J, Wolterbeek HT.** 1992. Analysis of major tomato xylem organic acids and PITC-derivatives of amino acids by RP-HPLC and UV detection. Plant and Soil **142:**81-89.
6. **Dalsing BL, Truchon AN, Gonzalez-Orta ET, Milling AS, Allen C.** 2015. Inorganic nitrogen reduction and detoxification are necessary for full *Ralstonia solanacearum* virulence on tomato. mBio **6:**e02471-02414.
