# Supplementary material for: A Single Regulator Mediates Strategic Switching between Attachment/Spread and Growth/Virulence in the Plant Pathogen Ralstonia solanacearum
Source: mBio. 2017 Sep 26;8(5):e00895-17. doi: 10.1128/mBio.00895-17 (PMC5615195; doi:10.1128/mBio.00895-17)
Supplement: TABLE S4 [file mbo005173501st4.pdf]

Table S4. **Strains, plasmids and primers used in this study**

Strains	Relevant characteristics	Source or reference
E.coli TOP10	F^- *mcrA* Δ(*mrr-hsdRMS-mcrBC*) Φ80*lacZ* ΔM15 Δ*lac*X74 *recA1 araD139* Δ(*ara-leu*)*7697 galU galK rpsL* (Str^r) *endA1 nupG*	Invitrogen
R. solanacearum		
GMI1000	Wild-type tomato isolate; Phylotype I seq 18 strain	(1)
Δ*phcA*	GMI1000 Δ*phcA*::Gm, Gm^r	This study
Δ*norB*	GMI1000 Δ*norB*::Gm, Gm^r	(2)
Δ*phcA*+*phcA*	GMI1000 Δ*phcA*::Gm; Gm^r + *phcA*pRCT*; Tet^r	This study
K60	Wild-type tomato isolate; Phylotype IIA seq 7 strain	(3)
Δ*phcA*	K60Δ*phcA*::Gm, Gm^r	Allen lab
Plasmids		
pRCT-GWY	Gateway destination vector with GMI1000 genome integration sites; Tet^r	(4)
*phcA*pRCT	Complement vector with PCR amplicon containing *phcA* cloned between Kpn1 and Xba1, after the removal of GWY cassette; Tet^r	This study
**Primers**		
phcA comp Kpn1	CGTA<u>GGTACC</u>GATCGATGTGATCCGGCCT	This study
phcA comp Xba1	CTGA<u>TCTAGA</u>CGTTTTCGTTGGAGGAGCG	This study
**qPCR primers**		
Bacterial genes		
hrpB-F	TCGACCCAACGCGGCAAGTC	This study
hrpB-R	CGAATGGCGGATCAGGCGCT	This study
aniA-F	CTTGGTGTACTGCTGACGAG	This study
aniA-R	CCTTCGGAGATCGGCATTT	This study
narG-F	GGTGTGCCTGTCGTTCTATG	This study
narG-R	TAGCGGACTTCGGTGTAGAA	This study
epsB-F	GACCGATGCCAACGCCGAG	This study
epsB-R	CGCGCCGATCAGCGGAAAGT	This study
serC-F	GGATGACGCGGCTTACGT	This study
serC-R	TCAACGCCGACGATGGT	This study
tek-F	ACTTTGATCCGTGCCCACTT	This study
tek-R	GCACGGGCAATTTCACCTTT	This study
fliC-F	TGCGAACTCGTACCTGCAAA	This study
fliC-R	ATGTTCTTGTTGGCCGTTGC	This study

rplM-F	CCGCAAAGCCCCATGAG	(5)
rplM-R	TGTCCGTCGCGTCAATCA	(5)
Plant defense genes		
PR-1a-F	TCA AAG AGC TGA TGA CTG TG	(6)
PR-1a-R	GTA CCA TTG CTT CTC ATC GT	(6)
ACO5-F	AGATGGGCATTGGGTGAACA	Allen lab
ACO5-R	TTCAGCCATCACTCGGTGTC	Allen lab
Pin2-F	TGATGCCAAGGCTTGTACTAGAGA	(6)
Pin2-R	AGCGGACTTCCTTCTGAACGT	(6)
Actin-F	TCAGCAACTGGGATGATATG	(6)
Actin-R	TTAGGGTTGAGAGGTGCTTC	(6)

* A single copy of complementing DNA was integrated into the *Rs* genome at the selectively neutral *att* site distant from deletion construct integration (4). Gm^r, gentamicin resistance; Tet^r, tetracycline resistance. Restriction endonuclease sites are underlined.

References

1. **Lugtenberg BJJ, Kravchenko, L.V., and Simons, M.** 1999. Tomato seed and root exudate sugars: composition, utilization by *Pseudomonas* biocontrol strains and role in rhizosphere colonization. Environmental Microbiology **1:**439-446.
2. **Dalsing BL, Truchon AN, Gonzalez-Orta ET, Milling AS, Allen C.** 2015. Ralstonia solanacearum Uses Inorganic Nitrogen Metabolism for Virulence, ATP Production, and Detoxification in the Oxygen-Limited Host Xylem Environment. Mbio **6**.
3. **Kelman A.** 1954. The relationship of pathogenicity of *Pseudomonas solanacearum* to colony appearance in tetrazolium medium. Phytopathology **44:**693-695.
4. **Monteiro F, Solé M, Dijk Iv, Valls M.** 2012. A chromosomal insertion toolbox for promoter probing, mutant complementation, and pathogenicity studies in *Ralstonia solanacearum*. Mol Plant-Microbe Interact **25:**557-568.
5. **Monteiro F, Genin S, vanDijk I, Valls M.** 2012. A luminescent reporter evidences active expression of *Ralstonia solanacearum* type III secretion system genes throughout plant infection. Microbiology **158:**2107-2116.
6. **Milling AS, Babujee L, Allen C.** 2011. *Ralstonia solanacearum* extracellular polysaccharide is a specific elicitor of defense responses in wilt-resistant tomato plants. PLoS One **6:**e15853.
