# Supplementary material for: A Single Regulator Mediates Strategic Switching between Attachment/Spread and Growth/Virulence in the Plant Pathogen Ralstonia solanacearum
Source: mBio. 2017 Sep 26;8(5):e00895-17. doi: 10.1128/mBio.00895-17 (PMC5615195; doi:10.1128/mBio.00895-17)
Supplement: TABLE S5 [file mbo005173501st5.pdf]

Table S5: List of *R. solanacearum* traits expressed differently *in vitro* and *in planta*

Previously Known Phenotypes	Expression in culture or *in planta*	Based on *in planta* RNA seq data
Swimming motility	not regulated by PhcA[1, 2], repressed by PhcA[3]	No change in expression levels of motility genes
EPS	Positively regulated by PhcA *in culture* [4], Positively regulated by PhcA *in planta*[5]	Positively regulated by PhcA
T3SS	Repressed by PhcA in culture[6], not repressed by PhcA *in planta* [7, 8]	*hrcC*, *hrpB*, *popA*, *popB* and *popC* are positively regulated by PhcA
Iron/siderohore	Repressed by PhcA[9]	same
SolIR QS system	Positively regulated by PhcA[10]	same
Cellulase, endo glucanase	Positively regulated[4, 11]	same
Ralfuranone synthesis	Positively regulated by PhcA[12]	same
TEK production	Induced by PhcA[4]	same
Twitching motility	*pilA* expression is repressed PhcA via PehR[13]	not affected

New Phenotypes	Phenotypes known in culture or *in planta*	Based on *in planta* RNA seq data
T6SS	not known	Positively regulated by PhcA
Nitrogen metabolism	denitrification genes are activated in planta[7]	Repressed by PhcA
Rhs	not known	3 of the rhs genes expression were down regulated
Hemagluttinin	not known	differentially regulated
Chemotaxis	not known	*cheY* and *cheD2* downregulated in *phcA* mutant
Autolysin	not known	not affected
Biofilm formation	reduced in *phcA* mutant	*phcA* mutant forms"Mat" like structure near the point of infection
Sulfur metabolism	Not known	Repressed by PhcA
Carbon metabolism	Not known	Pyurvate metabolism and TCA cycle upregulated

References

1. **Brumbley SM, Denny TP**. 1990. Cloning of wild-type *Pseudomonas solanacearum phcA*, a gene that when mutated alters expression of multiple traits that contribute to virulence. J Bacteriol.;**172**:5677-85.
2. **Tans-Kersten J, Brown D, Allen C**. 2004. Swimming motility, a virulence trait of *Ralstonia solanacearum*, is regulated by FlhDC and the plant host environment. Mol Plant Microbe Interact.**17**:686-95.
3. **Clough SJ, Flavier AB, Schell MA, Denny TP**. 1997.Differential Expression of Virulence Genes and motility in *Ralstonia* (Pseudomonas) *solanacearum* during exponential growth. Appl Environ Microbiol.**63**:844-50.
4. **Brumbley SM, Carney BF, Denny TP.** 1993. Phenotype conversion in *Pseudomonas solanacearum* due to spontaneous inactivation of PhcA, a putative LysR transcriptional regulator. J Bacteriol.**175**:5477-87. .
5. **McGarvey JA, Denny TP, Schell MA**. 1999. Spatial-Temporal and quantitative analysis of Growth and EPS I Production by *Ralstonia solanacearum* in resistant and susceptible tomato cultivars. Phytopathology. **89**:1233-9.
6. **Genin S, Brito B, Denny TP, Boucher C**. 2005. Control of the *Ralstonia solanacearum* Type III secretion system (Hrp) genes by the global virulence regulator PhcA. FEBS Lett. **579**:2077-81.
7. **Jacobs JM, Babujee L, Meng F, Milling A, Allen C**. 2012.The *in planta* transcriptome of *Ralstonia solanacearum*: conserved physiological and virulence strategies during bacterial wilt of tomato. MBio.**3**.
8. **Monteiro F, Genin S, vanDijk I, Valls M**. 2012. A luminescent reporter evidences active expression of *Ralstonia solanacearum* type III secretion system genes throughout plant infection. Microbiology.**158**:2107-16.
9. **Bhatt G, Denny TP.** 2004. *Ralstonia solanacearum* iron scavenging by the siderophore staphyloferrin B is controlled by PhcA, the global virulence regulator. J Bacteriol.**186**:7896-904.
10. **Flavier AB, Schell MA, Denny TP**. 1998. An RpoS (sigmaS) homologue regulates acylhomoserine lactone-dependent autoinduction in *Ralstonia solanacearum*. Mol Microbiol.**28**:475-86.
11. **Schell MA, Denny TP, Huang J.** 1994. Extracellular virulence factors of *Pseudomonas solanacearum*: role in disease and regulation of expression. In *Molecular Mechanisms of Bacterial Virulence*, ed. C Kado, J Crosa, pp. 311–324. Dordrecht: Kluwer
12. **Schneider P, Jacobs JM, Neres J, Aldrich CA, Allen C, Nett M**, et al. 2009.The global virulence regulators VsrAD and PhcA control secondary metabolism in the plant pathogen *Ralstonia solanacearum*. ChemBioChem.**10**.
13. **Kang Y, Saile E, Schell MA, Denny TP**. 1999. Quantitative immunofluorescence of regulated eps gene expression in single cells of *Ralstonia solanacearum*. Appl Environ Microbiol.**65**:2356-62.
